# Functional parcellation of human and macaque striatum reveals human-specific connectivity in the dorsal caudate

**DOI:** 10.1016/j.neuroimage.2021.118006

**Published:** 2021-07-15

**Authors:** Xiaojin Liu, Simon B. Eickhoff, Svenja Caspers, Jianxiao Wu, Sarah Genon, Felix Hoffstaedter, Rogier B. Mars, Iris E. Sommer, Claudia R. Eickhoff, Ji Chen, Renaud Jardri, Kathrin Reetz, Imis Dogan, André Aleman, Lydia Kogler, Oliver Gruber, Julian Caspers, Christian Mathys, Kaustubh R. Patil

**Affiliations:** a Institute of Neuroscience and Medicine (INM-7), Heinrich Heine University Düsseldorf, Düsseldorf, Germany; b Institute of Systems Neuroscience, Research Centre Jülich, Jülich, Germany; c Institute of Neuroscience and Medicine (INM-1), Research Centre Jülich, Jülich, Germany; d Institute for Anatomy I, Medical Faculty, Heinrich Heine University Düsseldorf, Düsseldorf, Germany; e Wellcome Centre for Integrative Neuroimaging, Centre for Functional MRI of the Brain (FMRIB), Nuffield Department of Clinical Neurosciences, John Radcliffe Hospital, University of Oxford, Oxford, United Kingdom; f Donders Institute for Brain, Cognition and Behaviour, Radboud University Nijmegen, Nijmegen, Netherlands; g Department of Biomedical Sciences of Cells & Systems, University Medical Center Groningen, Groningen, Netherlands; h Institute of Clinical Neuroscience and Medical Psychology, Medical Faculty, University of Düsseldorf, Düsseldorf, Germany; i Division of Psychiatry, University of Lille, CNRS UMR9193, SCALab & CHU Lille, Fontan Hospital, CURE platform, Lille, France; j JARA-BRAIN Institute Molecular Neuroscience and Neuroimaging, Forschungszentrum Jülich, RWTH Aachen University, Aachen, Germany; k Department of Neurology, RWTH Aachen University, Aachen, Germany; l Department of Neuroscience, University Medical Center Groningen, University of Groningen, Groningen, Netherlands; m Department of Psychiatry and Psychotherapy, Medical School, University of Tübingen, Germany; n Section for Experimental Psychopathology and Neuroimaging, Department of General Psychiatry, Heidelberg University, Germany; o Department of Diagnostic and Interventional Radiology, Medical Faculty, University of Düsseldorf, Düsseldorf, Germany; p Research Center Neurosensory Science, Carl von Ossietzky Universität Oldenburg, Oldenburg, Germany; q Institute of Radiology and Neuroradiology, Evangelisches Krankenhaus, University of Oldenburg, Oldenburg, Germany

**Keywords:** Non-human primate, Cross-species comparison, Striatum, Connectivity-based parcellation, Parkinson's disease, Schizophrenia

## Abstract

A wide homology between human and macaque striatum is often assumed as in both the striatum is involved in cognition, emotion and executive functions. However, differences in functional and structural organization between human and macaque striatum may reveal evolutionary divergence and shed light on human vulnerability to neuropsychiatric diseases. For instance, dopaminergic dysfunction of the human striatum is considered to be a pathophysiological underpinning of different disorders, such as Parkinson's disease (PD) and schizophrenia (SCZ). Previous investigations have found a wide similarity in structural connectivity of the striatum between human and macaque, leaving the cross-species comparison of its functional organization unknown. In this study, resting-state functional connectivity (RSFC) derived striatal parcels were compared based on their homologous cortico-striatal connectivity. The goal here was to identify striatal parcels whose connectivity is human-specific compared to macaque parcels. Functional parcellation revealed that the human striatum was split into dorsal, dorsomedial, and rostral caudate and ventral, central, and caudal putamen, while the macaque striatum was divided into dorsal, and rostral caudate and rostral, and caudal putamen. Cross-species comparison indicated dissimilar cortico-striatal RSFC of the topographically similar dorsal caudate. We probed clinical relevance of the striatal clusters by examining differences in their cortico-striatal RSFC and gray matter (GM) volume between patients (with PD and SCZ) and healthy controls. We found abnormal RSFC not only between dorsal caudate, but also between rostral caudate, ventral, central and caudal putamen and widespread cortical regions for both PD and SCZ patients. Also, we observed significant structural atrophy in rostral caudate, ventral and central putamen for both PD and SCZ while atrophy in the dorsal caudate was specific to PD. Taken together, our cross-species comparative results revealed shared and human-specific RSFC of different striatal clusters reinforcing the complex organization and function of the striatum. In addition, we provided a testable hypothesis that abnormalities in a region with human-specific connectivity, i.e., dorsal caudate, might be associated with neuropsychiatric disorders.

## 1 Introduction

Animal models provide important perspectives on neural functions, structures and disease, with several studies reporting an overall functional and structural similarity between non-human primates and humans (Goulas et al., 2014; Mars et al., 2011, 2018; Miranda-Dominguez et al., 2014; van den Heuvel et al., 2019). However, the same studies have also reported region-specific divergences revealing human-specific features potentially related to human-specific disorders. Consequently, comparing brain organization between humans and non-human primates can elucidate differentiation in brain organization potentially rooted in the process of species evolution and enrich our understanding of specializations in human brain organization (de Schotten et al., 2019; Rilling, 2014; Van den Heuvel et al., 2016) [also see: Friedrich (2021) in this issue].

The striatum is a crucial component of the basal ganglia which works in concert with the cerebral cortex to plan and execute behaviors (Haber et al., 2006; Haber and Knutson, 2010; Marquand et al., 2017; Smeets et al., 2000). Through diverse afferent projections from the cerebral cortex, the striatum is embedded in multiple basal ganglia circuits and mediates motivations and emotions that drive planning, cognition that generate appropriate strategy, and action execution (Haber, 2003; Nakano et al., 2000). The classical understanding of functional and structural organization of the striatum is primarily derived from anatomical and physiological findings in macaques (Alexander and Crutcher, 1990; Alexander et al., 1986; Künzle, 1975; Selemon and Goldman-Rakic, 1985). The internal capsule was artificially considered as a functional and structural boundary separating the striatum into caudate and putamen with the caudate considered to be primarily involved in cognition while the putamen in motor-related functions. However, functional and structural complexity of the striatum based on cortico-striatal circuits goes beyond this simplistic demarcation as demonstrated by several studies in macaque monkeys (Calzavara et al., 2007; Ferry et al., 2000; Yeterian and Pandya, 1991). For instance, the head and the tail circuits of the caudate are involved in short-term and long-term valuation, respectively (Hikosaka et al., 2014). Previous studies in macaques have shown that through integrating various information within the cortico-striatal circuits, both the caudate and the putamen participate directly in reward guided behavior and learning (Apicella et al., 2011; Hassani et al., 2001; Hikosaka et al., 2014, 1989; Histed et al., 2009; Watanabe et al., 2003) and the cortico-striatal reward circuitry structure and function is conserved across humans and primates (Haber and Knutson, 2010). In primates, projections from anteromedial prefrontal cortex to dorsoanterior striatum mediates learning processes related to reward-related actions, while projections from sensorimotor cortex to dorsoposterior striatum mediated processes related to acquisition of habits (Balleine et al., 2007). These projections route the information flow through substantia nigra pars reticularis (SNr) and globus pallidus internal segment (GPi) and back to the cerebral cortex. This prior knowledge from non-human primates and subsequent in-vivo neuroimaging findings in humans suggests that the human striatum is potentially divided into several structural and functional subregions (i.e. parcels) based on their involvement in multiple cortico-striatal circuits (Choi et al., 2012; Leh et al., 2007; Tziortzi et al., 2014).

Furthermore, recent studies have provided evidence for a correspondence between cortico-striatal functional networks and their gene expression profiles (Anderson et al., 2018) which combined with the lack of human-specific transcriptional signature of neoteny in the striatum (Bakken et al., 2016) suggest a strong evolutionarily conserved genetic makeup of the human striatum. However, recent studies have identified a substantial number of differentially expressed genes primarily related to dopamine biosynthesis in the human striatum compared to other species (Raghanti et al., 2016; Sousa et al., 2017). These evidences further support our investigation in shared and human-specific functional organization of the striatum.

Due to its key roles in cognitive, emotional, executive and motor functions, the human striatum has been implicated in the pathophysiology of several diseases. Among them, Parkinson's disease (PD) (Owen et al., 1992; Zhai et al., 2018) and schizophrenia (SCZ) (Li et al., 2020; Simpson et al., 2010) are two major socio-economically relevant disorders with a clear link to dopaminergic deficits within the striatum. Striatal functional and structural abnormalities have been found in patients compared to healthy controls (HC) (He et al., 2019; Xu et al., 2016). Generally, animal models provide relevant insight into potential pathophysiological mechanisms, options for medical treatment and clinical applications of such experiments for these neuropsychiatric diseases (Choudhury and Daadi, 2018; Qiu et al., 2019). Especially, rodent and macaque models are widely used to investigate these disorders (Castner et al., 2004; Cenci and Crossman, 2018), however, animal models are insufficient if they diverge from human brain organization. Furthermore, many neuropsychiatric diseases primarily affecting humans might be influenced by various factors specific to humans like, personality, family and social environment which in turn are associated with brain organization. It is not possible to transfer such complex characteristics and their interactions with brain organization to animal models. Hence, it is necessary to investigate whether functional and structural abnormalities of the striatal organization specific to humans are related to neuropsychiatric diseases.

Recent advances in non-invasive *in-vivo* neuroimaging techniques in humans and non-human primate makes direct cross-species comparison possible (de Schotten et al., 2019) (also see In this issue: Friedrich et al.). Comparison of the structural organization of the cortico-striatal circuits between human and macaque striatum have been conducted using probabilistic diffusion tractography (PDT) based on diffusion MRI (Neggers et al., 2015; Xia et al., 2019). Neggers et al. (2015) compared connections between cortical motor areas and the striatum in human and macaque using PDT. They found that the frontal eye fields (FEF) connected with the head of the caudate and anterior putamen, and the primary motor cortex (M1) connected with more posterior parts of the caudate and putamen in macaque. However, in human, the connectivity of FEF and M1 is largely with the posterior putamen and to a smaller degree with the caudate. Xia et al. (2019) also used PDT to identify the ventral striatum in humans and macaques based on their cortico-striatal structural connectivity, and then examined interspecies differences in the structural connectivity fingerprints of this region. These results show that the structural connectivity for subregions of the ventral striatum might be dissimilar between humans and macaques.

Although previous studies have provided rich and diverse information about cortico-striatal structural connectivity (Neggers et al., 2015; Xia et al., 2019), little is known about the functional connectivity (FC) of the macaque striatum and whether it differs from humans. FC uses correlation of time series from blood oxygenation level dependent (BOLD) functional MRI signals, which reflects the temporal synchrony of neuronal activation patterns between brain regions [for review, see Van Den Heuvel and Pol (2010)] including when the FC is measured at rest—i.e. resting-state FC (RSFC) (Biswal et al., 2010). RSFC has been widely used in human research to investigate intrinsic neuronal activation pattern of the striatum to reveal its functional organization (Barnes et al., 2010; Janssen et al., 2015; Jaspers et al., 2017; Jung et al., 2014). For instance, Jung et al. (2014) examined the parcellation of the human striatum based on its RSFC to the whole brain. Primarily, the caudate is divided into three subregions along the anterior-ventral axis and the posterior-dorsal axis. The spatial pattern of these three clusters correspond to the head, body, and tail of the caudate. Similarly, the putamen was split into three subregions but along an anterior-posterior axis. In our own work (Liu et al., 2020), we identified joint multi-modal parcellation of the striatum which included RSFC as one modality. We found that the striatum was split into subregions along the rostro-caudal and ventro-dorsal axes from coarse (*k* = 3) to fine-grained (*k* = 9) parcellations based on its intrinsic RSFC. However, similarities and differences in the functional parcellation of the striatum between human and macaque are unclear. Such a cross-species analysis can shed light on similarities of human brain organization with our phylogenetically close relatives, and, more importantly, reveal organization specific to humans. In addition, it remains an open question whether the human-specific striatal organization is involved in complex neuropsychiatric disorders which are often specific to humans.

In this study, we capitalize on *in-vivo* neuroimaging data to directly compare the functional organization of the striatum between humans and macaques, and subsequently investigated cortico-striatal RSFC and structural alteration in the striatal subregions in two neuropsychiatric diseases (PD, SCZ). The human neuroimaging data for parcellation was assessed from the Human Connectome Project (HCP), while macaque neuroimaging data was assessed from the PRIMatE Data Exchange (PRIME-DE). There is a growing interest in comparative MRI investigation of functional and structural organization of the brain between humans and macaques (Balsters et al., 2020; Mars et al., 2018; Vanduffel et al., 2014; Xu et al., 2019). However, most previous studies only recruited very small samples (*n* < 10) of non-human primate subjects. Although technological and methodological advances have promoted the development of non-human primate research during the past two decades, neuroimaging data collection remained limited due to lack of necessary facilities and capabilities. PRIME-DE addressed these challenges by aggregating independently acquired non-human primate MRI datasets and openly sharing them through the International Neuroimaging Data-sharing Initiative (INDI) (Milham et al., 2020, 2018). Benefiting from these two open datasets, we directly compare functional aspects of brain organization between human and macaque. To compare functional organization of human and macaque striatum, we first employed connectivity-based parcellation (CBP) [for review, see Eickhoff et al. (2015), Eickhoff et al. (2018)] that can identify subregions based on their similarities in connectivity, such as RSFC and PDT (Genon et al., 2018; Plachti et al., 2019a). We used RSFC as connectivity measure to separately perform CBP in human and macaque, and then compared the resulting parcels based on their connectivity to a set of homologous cerebral regions. The Pearson correlation distance was used to estimate the dissimilarity of connectivity fingerprints between human and macaque striatal subregions. Finally, we compared difference in cortico-striatal RSFC and in a cross-modal analysis also used voxel-based morphometry (VBM) to investigate functional and structural alterations in the human striatal subregions between patients (PD, SCZ) and healthy controls (HC).

## 2 Methods

**Fig. 1:**
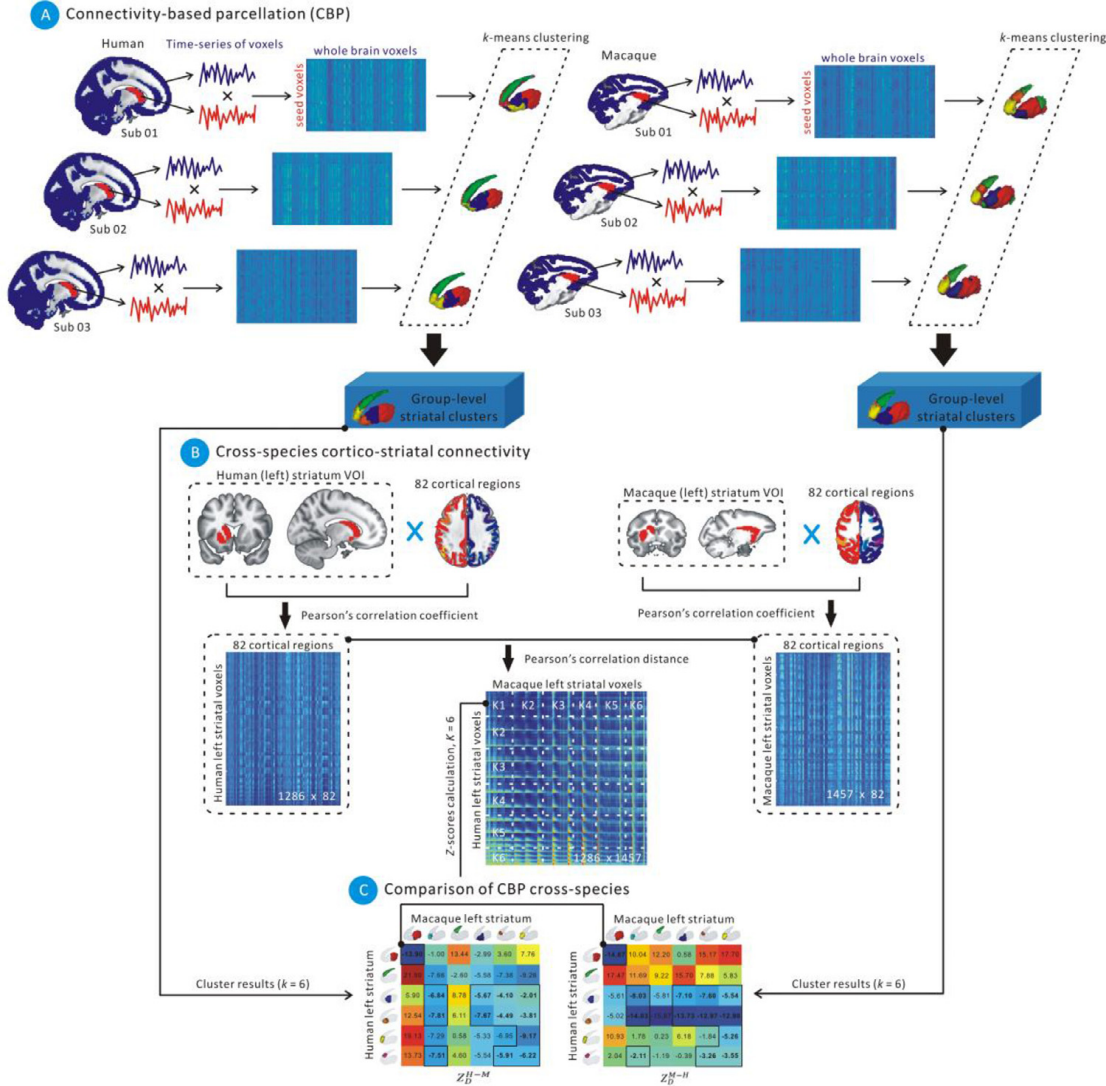
A sketch of our proposed pipeline depicting its use on the left striatum. (A) Connectivity-based parcellation (CBP) of human and macaque striatum based on the RSFC between the striatal voxels and whole-brain gray matter voxels. Each subject's RSFC connectivity matrix is then subjected to *k*-means clustering while varying number of clusters. The final number of clusters was selected using several data-driven model selection criteria. The subject-level clusters where then aggregated into group-level clustering for humans and macaques separately. (B) Cross-species cortico-striatal connectivity: The RSFC between striatal voxels and regions of regional map (RM) was calculated for each subject and then averaged. The distance between each human striatum voxel and each macaque striatum voxel was calculated using the Pearson correlation (1-R) between the corresponding RM connectivity profiles. (C) Comparison of CBP-derived parcels cross-species. The permutation based *Z*-scores were calculated for comparing the cortico-striatal RSFC of the CBP-derived parcels (shown here for *k* = 6).

Briefly, we followed a two-step procedure (see Fig. 1). In the first step we performed CBP based on the RSFC of the striatal voxels with the whole-brain gray matter voxels to uncover the functional organization of the human and macaque striatum separately and chose parcellation schemes based on data-driven model selection. In the second step, we performed cross-species comparison of the striatal clusters from the first step based on their connectivity with a set of homologous cortical regions. The striatal clusters were then investigated for differences between patients (PD and SCZ) and HC in their cortico-striatal RSFC and structural atrophy based on gray matter (GM) volume.

### 2.1 Dataset

For CBP, both human and macaque resting-state functional magnetic resonance imaging (fMRI) datasets were obtained from open access sources. We used the Human Connectome Project (HCP) young adult sample (https://www.humanconnectome.org), assessing 324 unrelated subjects (male/female: 158/166, age range: 22–37 years). We also selected another dataset with 206 unrelated subjects (male/female: 100/106, age range: 22–36 years) from HCP in order to retest functional parcellation results (Supplementary Material). The macaque monkey dataset was obtained from the recently established PRIMatE Data Exchange (PRIME-DE) project (http://fcon_1000.projects.nitrc.org/indi/indiPRIME.html). PRIME-DE currently contains 219 macaque monkeys from 25 institutes. In this study, we selected 56 macaque subjects from four institutes; University of Oxford (male/female: 20/0, age range: 2.41–6.72 years), Institute of Neuroscience, China (male/female: 7/1, age range: 3.80–5.99 years), Newcastle University (male/female: 12/2, age range: 3.90–13.14 years) and University of California, Davis (male/female: 0/19, age range: 18.50–22.50 years). We only used these four macaque datasets because they provide relatively large resting-state fMRI data sample sizes. Details of scanning and imaging of HCP and PRIME-DE datasets are listed in Table S1 and Table S2. Given all macaque data contributed to PRIME-DE project were made available regardless of data quality, we performed quality control and excluded 7 macaque subjects before data analysis (Supplementary Materials).

For the clinical datasets, we collected resting-state fMRI and T1-weighted images of Parkinson's disease (PD) patients from Heinrich Heine University Düsseldorf and RWTH Aachen University (Pläschke et al., 2017). Together, these two datasets included 101 patients (female: 47, age: 63.09 ± 10.06) and 96 healthy controls (HC, female: 45, age: 58.87 ± 9.81). We collected resting-state fMRI of schizophrenia (SCZ) patients and controls from RWTH Aachen University, Center for Biomedical Research Excellence (Mayer et al., 2013), the University of Groningen (Chen et al., 2020a, 2020b; Vercammen et al., 2010), the University of Göttingen, the University of Lille (Lefebvre et al., 2016), and Utrecht University (Clos et al., 2014). The pooled SCZ dataset included 142 patients (female: 41, age: 34.94 ± 11.72) and 136 HC (female: 40, age: 33.82 ± 11.11). We also collected T1-weighted structural MRI data of SCZ patients from the Center for Biomedical Research Excellence, the University of Groningen, the University of Lille, the Technical University of Munich and Utrecht University. These dataset totally included 159 patients (female: 54, age: 35.92 ± 12.08) and 166 HC (female: 64, age: 34.32 ± 11.94). No significant sex difference was observed between patients and HC [$\chi^{2}$-test: *p* = 0.96 for PD and HC, *p* = 0.92 for SCZ and HC (resting-state fMRI datasets), *p* = 0.39 for SCZ and HC (T1-weighted images datasets)]. A significant difference was found in age between PD and HC (two-sample *t*-test *p* < 0.01), but not between SCZ and HC (*p* = 0.41 for resting-state fMRI datasets, *p* = 0.23 for T1-weighted images datasets). Additional information on MR scanning parameter for above datasets can be found in Supplementary Materials.

For PRIME-DE, all experimental procedures were approved by local ethics boards prior to any data collection. UK macaque datasets were obtained with Home Office approval and abide with the European Directive on the protection of animals used in research (2010/63/EU). For the NIN Primate Brain Bank/Utrecht University dataset, postmortem specimens were loaned from the Netherlands Institute of Neuroscience Primate Brain Bank (PBB; http://www.primatebrainbank.org/). No individuals were sacrificed for PBB brain issue. Instead, brains were collected from individuals that died from natural causes or that had to be humanely euthanized for reasons unrelated to the tissue collection.

The ethics protocols for analyses of these data were approved by the Heinrich Heine University Düsseldorf ethics committee (No. 4039, 4096).

### 2.2 Data preprocessing

Human resting-state fMRI data preprocessing was performed using SPM12 (Wellcome Trust center for Neuroimaging, London, http://www.fil.ion.ucl.ac.uk/spm/software/spm12). The preprocessing was consistent across the human datasets. For the HCP we used the minimally preprocessed volumetric data in the Montreal Neurological Institute (MNI) space (Glasser et al., 2013; Van Essen et al., 2012). We performed additional steps on HCP data and the other human data sets were preprocessed to optimally align fMRI time-series in the common MNI space and remove motion artifacts and nuisance signals. For each subject, we excluded the first four echo planar imaging (EPI) volumes to allow the MRI signal to reach steady state followed by realignment to the first and successively to the mean image. Next, the mean EPI was coregistered to the GM probability map and normalized to MNI space using the unified segmentation algorithm (Ashburner and Friston, 2005). Subsequently, we applied this non-linearly transformation to all EPI images before smoothing with a kernel of 5 mm full width at half maximum (FHWM). Finally, we performed time series denoising using multiple regression of mean white matter (WM) and cerebrospinal fluid (CSF) signals, and 24 motion parameters (Satterthwaite et al., 2013) before band-pass filtering (0.01–0.08 Hz) the residuals. Note that we did not use the individual T1 images for normalization as the EPI based alignment has been shown to provide good results (Calhoun et al., 2017; Dohmatob et al., 2018), and the segmentation of the mean EPIs with unified segmentation uses tissue probability maps as priors helping delineation in sub-cortical regions.

Macaque resting-state fMRI data preprocessing was performed using SPM12 and the FMRIB Software Library (FSL, https://fsl.fmrib.ox.ac.uk/fsl/fslwiki). The first four volumes of the EPI images were discarded. After brain extraction using FSL BET, all images were head motion corrected by aligning to the first EPI image. Next, T1 image was registered to Yerkes19 template, and the mean EPI image was coregistered to T1 image. The EPI images were non-linearly normalized to Yerkes19 template with voxel size of $1\times 1\times 1mm^{3}$ by using the transformation parameters from last step in FSL. Then all EPI images were smoothed with a FHWM of 3 mm. Next we performed WM, CSF signal and head motion regression as well as band-pass filtering similar to the human data.

We also tested the robustness of our human CBP results using the FMRIB's ICA-based Xnoiseifier (FIX) based artifact removal on the HCP data. For all human and macaque subjects, we further calculated voxel-wise temporal signal-to-noise (tSNR) for all the voxels in the striatum (Supplementary Materials).

### 2.3 Region of interest (ROI) definition

*Human striatum:* The region of Interest (ROI) for the human left and the right striatum were extracted using the Harvard-Oxford subcortical structural probability atlas available via FSL. We extracted the caudate and putamen with a voxel size of 2 mm x 2 mm x 2 mm based on a probability threshold of 25%, and then combined these structures into one human striatal ROI for each hemisphere. This procedure resulted in a left striatum ROI with 1286 voxels (caudate: 487, putamen: 799) and a right striatum ROI comprising 1307 voxels (caudate: 511, putamen: 796).

*Macaque striatum:* The ROI for the macaque left and right striatum were extracted using the INIA19 template (Rohlfing et al., 2012). We extracted both caudate and putamen and resampled them to the Yerkes19 space with a voxel size of 1 mm x 1 mm x 1 mm. We also combined the caudate and putamen into one macaque striatal ROI for each hemisphere. The numbers of voxels was 1457 (caudate: 551, putamen: 906) in the left and 1445 (caudate: 550, putamen: 895) in the right striatum of the macaques. Different voxel sizes resulted in a similar resolution for each species as reflected in the similar number of striatal voxels in human and macaque ROIs.

### 2.4 Connectivity-based parcellation using functional connectivity

#### 2.4.1 Whole-brain resting-state functional connectivity

We estimated the resting-state functional connectivity (RSFC) between the striatal voxels and the whole-brain voxels. To this end, we used preprocessed resting-state fMRI data to calculate the Pearson correlation between the time series of each voxel within the striatum and all other GM voxels for each human and macaque subject. The correlation coefficients were then Fisher-*Z* transformed. One resting-state functional connectivity matrix was calculated per individual.

#### 2.4.2 Clustering algorithm

In line with previous connectivity-based parcellation (CBP) studies (Crippa et al., 2011; Genon et al., 2018; Jung et al., 2014; Plachti et al., 2019a; Xu et al., 2020a), voxels within a ROI are grouped into distinct clusters (i.e., subregions) through a clustering algorithm based on their similarity in RSFC patterns. Generally, *k*-means clustering divides a given ROI into a preselected number of *k* non-overlapping clusters (Nanetti et al., 2009). The *k*-means algorithm is known to provide accurate parcellation results compared to other clustering methods (Thirion et al., 2014). In this study, we applied the *k*-means clustering as implemented in the yael package (https://gforge.inria.fr/projects/yael) on the individual RSFC matrix. The numbers of potential subdivisions from 2 to 7 clusters were investigated. Based on reported results in the literature, we assumed that a meaningful organization of the striatum can be observed at low and medium resolution but not at very high resolution (i.e. not in more than 7 subdivisions). For each *k*-means run, the best solution based on the sum of squares from 100 initializations with a randomly placed initial cluster centers were used. Importantly, for each solution, *k*-means clustering was performed at the individual level. Resulting individual-level cluster solutions were then combined into a single group-level parcellation by computing the most frequent cluster assignment for each striatal voxel across all human or macaque subjects separately. Note that we use the terms cluster and parcel interchangeably.

The group-level parcellation from the individual-level clusters were calculated as follows. First, a consensus clustering of the individual-level clustering solutions is calculated using the hierarchical clustering algorithm with the Hamming distance to account for the arbitrariness of the cluster ids across solutions and with the number of clusters equal to the number of clusters in the individual solutions under consideration. Each individual-level solution is then matched with the hierarchical clustering using a permutation that maximizes the match of the cluster ids. The final group-level solution is then calculated as the mode of the aligned individual-level clustering solutions. This procedure identifies a clustering that is representative of the whole group.

#### 2.4.3 Cluster selection criteria

As clustering is an unsupervised process, it is difficult to know which model selection criteria to use (Friedman et al., 2001). We, therefore, selected the cluster solutions based on five different criteria (Eickhoff et al., 2015): two topological criteria (percentage of misclassified voxels, and hierarchy index), an information-theoretic criterion (variation of information across cluster solutions) and two cluster separation criteria (change in inter/intra cluster distance and the silhouette index). Detailed information about each criterion and the selection procedure can be found in the Supplementary Materials.

### 2.5 Cross-species comparison

#### 2.5.1 Homologous cortico-striatal RSFC

We calculated cortico-striatal RSFC of humans and macaques based on the cortical ROIs selected from the Regional Map parcellation (RM) defined by Kötter and Wanke (2005). The RM is based on cytoarchitectonic, gross anatomical, and functional criteria, minimizing cross-species discrepancies in ontology. Reid et al. (2016) compared diffusion-based structural connectivity strength in humans with neuronal tracer-based structural connectivity strength in macaques based on the RM showing a moderately high correspondence. Goulas et al. (2014) employed the RM to compare the structural connectivity between macaques and humans and demonstrated a good overall correspondence providing additional validation of the RM for cross-species comparison. The RM thus provides a good way for cross-species comparison between humans and macaque monkey. The RM parcellation contains 82 cortical regions (see Fig. S1 and Table S3). For each human and macaque subject, we calculated the Pearson correlation between the averaged time series across all voxels within each cortical RM ROI and the time series of each striatal voxel to create a cortico-striatal RSFC matrix. Each row of this matrix represents the connectivity pattern of a striatal voxel with each of the 82 RM ROIs. We averaged the connectivity matrices across human subjects and across macaque subjects to generate group-representative homologous cortico-striatal RSFC matrices separately for both species and both hemispheres (Fig. 4).

#### 2.5.2 Cluster-based cross-species comparison

As the RM regions are considered homologous, we could estimate the dissimilarity between a human striatal voxel and a macaque striatal voxel using the Pearson correlation distance (1−r) between their connectivity pattern with the RM regions. By calculating distances between all pairs of human and macaque striatal voxels we obtained two cross-species distance matrices (*D*), one for the left and one for the right striatum. In this matrix an element $D_{ij}$ quantifies the dissimilarity between the cortico-striatal RSFC pattern of a human striatal voxel *i* with that of a macaque striatal voxel *j*. As our aim is to compare human and macaque striatal clusters obtained by functional CBP, we used a voxel-wise distance matrix *D* together with the cluster labels from the clustering results to estimate the distance between all human-macaque parcel pairs. The distance between a human-macaque parcel pair H-M was calculated as the average of all human and macaque striatal voxel-wise distances assigned the human cluster H and the macaque cluster M, i.e. $Mean_{true(H-M)}=\sum_{i\in H,j\in M}D_{ij}/\left|H\right|\times \left|M\right|$. A human-macaque parcel pair can be deemed to have a significantly similar cortico-striatal RSFC pattern if the distance between them is less than what can be expected by chance. To achieve this, we used a permutation test in which the cluster labels assigned to the voxels were permuted. As a human parcel can be similar to multiple macaque parcels and vice versa, we performed permutations in two ways. First, we test whether a human parcel is similar to randomly generated macaque parcel by shuffling the cluster assignment of macaque striatal voxels (the columns of *D*) and calculating the distance between a human parcel and the shuffled macaque parcel. By repeating this permutation 5000 times we obtain an empirical distribution [*Mean_1,_*_2_*_,_*_3… 5000_ *_(H_* *_− M)_*]. The mean and standard deviation of this empirical distribution is then used to calculate the *Z*-score of the true distance between parcels H and M:

$$Z_{D}^{H-M}=\frac{Mean_{true\left(H-M\right)}-Mean_{permuted\left(H-M\right)}}{Std_{permuted\left(H-M\right)}}$$

A lower value of $Z_{D}^{H-M}$ reflects higher similarity of the cortico-striatal RSFC between a human parcel H and a macaque parcel M compared to randomly selected macaque striatal voxels. In the same way, we calculate $Z_{D}^{M-H}$ by shuffling the cluster assignments of the striatal human voxels which reflects relative similarity of the cluster pair with respect to randomly selected human striatal voxels:

$$Z_{D}^{M-H}=\frac{Mean_{true\left(M-H\right)}-Mean_{permuted\left(M-H\right)}}{Std_{permuted\left(M-H\right)}}$$

We employed a strict criterion that both Z-scores must be below a threshold value to declare the corresponding cluster pair to have significantly similar cortico-striatal RSFC. We set the significance threshold at 2 standard deviations (2SD = −1.96).

In addition, we also visually checked spatial correspondence between the cluster pairs (i.e. within the whole striatum) and if a human striatal cluster was not deemed similar to any spatially corresponding macaque striatal cluster then this cluster was labeled as showing dissimilar cortico-striatal RSFC between human and macaque—i.e. a cluster with human-specific cortico-striatal RSFC. Macaque-specific clusters were identified in a similar way.

#### 2.5.3 Human-specific cortico-striatal RSFC

We further investigated how the cortico-striatal RSFC differed between humans and macaques for the human-specific striatal clusters identified in our previous analysis. For this, first the averaged cortico-striatal RSFC matrices based on each striatal voxel and the 82 cortical RM regions for all human and all macaque subjects were calculated. The average cortico-striatal RSFC across a given human-specific cluster voxels and corresponding spatially similar macaque-specific cluster voxels was calculated. The macaque-specific connectivity was subtracted from the human-specific connectivity and the difference was *Z*-scored for visualization purposes. Note that a higher score here indicates a stronger connectivity in human while a lower score indicates a stronger connectivity in macaque.

### 2.6 Cortico-striatal RSFC and voxel-based morphometry (VBM) analysis in disease

To gain insights into which striatal clusters, and especially the human-specific striatal clusters, are related to clinically relevant functional and structural alterations in humans, we investigated differences in cortico-striatal RSFC and GM volume of striatal clusters between patients (PD and SCZ) and HC.

Resting-state functional images were preprocessed with the same steps as described in “*Data preprocessing”*. We calculated the Pearson correlation between the averaged time series of the voxels within a given striatal cluster and averaged time series of all voxels within each cortical region based on RM. Finally, we examined differences in RSFC of each striatal cluster and each RM region between patients and HC by using a two-sample *t*-test while controlling for “sex”, “age” and “sites”. The resulting p-values were FDR corrected. This analysis was performed for left and right striatal clusters separately.

We also investigated differences in averaged GM volume of striatal cluster between patients and HC. T1-weighted images were preprocessed using the Computational Anatomy Toolbox (CAT12, http://www.neuro.uni-jena.de/cat/) in SPM12. All images were first segmented into GM, WM, and CSF using the standard unified segmentation. We then processed the images using the standard settings in CAT12, including DARTEL normalization, spatially adaptive non-linear means denoising, a Markov random field weighting of 0.15, bias regularization (0.0001) and FWHM cutoff (60 mm). The resulting normalized GM segments were modulated only for the non-linear components of the deformation, which means we only used local and non-linear deformations to adjust the head size to estimate the GM volume. Next, we extracted the average GM volume for the human-specific striatal clusters for each subject and examined if they differ between patients and HC. Considering that differences in GM volume might be associated with sex, age, hemisphere and sites, we applied a six-way analysis of variance (ANOVA) that included not only “disease status”, but also “striatal clusters”, “sex”, “age”, “hemisphere” and “sites” as factors.

## 3 Results

We first investigated the functional parcellation of the human and macaque striatum separately from low to high levels of subdivision and examined how the different cluster solutions were supported by the five data-driven model selection criteria. For each striatum ROI (human and macaque, left and right side), we identified the appropriate cluster solution that was supported by the majority of the criteria.

### 3.1 Human striatum

**Fig. 2:**
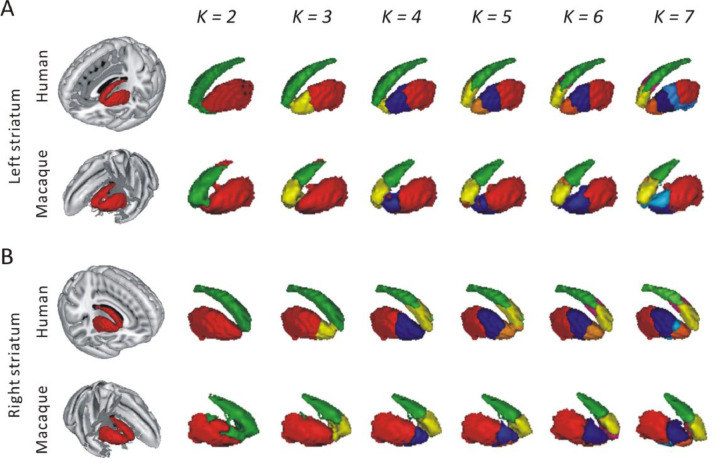
Connectivity-based parcellation (CBP) of left (A) and right (B) human and macaque striatum at different levels (*k*) of subdivision.

We found that the human striatum was split into clusters (i.e. parcels) along the dorso-ventral and rostro-caudal axis for both left and right side from low to high levels of subdivision (i.e. *k* = 2–7 clusters, Fig. 2). At *k =* 2, the human striatum was divided into caudate and putamen. At *k =* 3, the putamen was subdivided into a rostral and caudal cluster. At *k =* 4, for the left side, the putamen was further split into ventral, central and caudal clusters. However, for the right striatum, the caudate was split into rostral and dorsal cluster. At *k =* 5, the left caudate was divided into rostral and dorsal clusters, which similar to that of right caudate at *k =* 4. In turn, the right putamen was split into three clusters, which similar to that of left putamen at *k =* 4. Similar striatal clusters were found between left and right side at *k =* 6, including rostral, dorsomedial, dorsal caudate and ventral, central, caudal putamen. At the highest level of subdivision (*k =* 7), for the left side, the caudal putamen was divided into a dorsal and a ventral part. However, for the right side, we found an additional small cluster located between the central and ventral putamen.

### 3.2 Macaque striatum

As expected, at *k* = 2, both macaque left and right striatum were divided into caudate and putamen similarly to what we found in the human striatum (Fig. 2). At *k* = 3, the caudate was divided into rostral and dorsal parts, while at *k* = 4, we found a ventral cluster that was derived from the putamen in the 2-cluster solution. At *k* = 5, for the left side, the rostral caudate from the 3-cluster solution was split into two clusters including the medial and lateral parts. For right side, the ventral putamen obtained from *k =* 4 was divided into two parts along the dorso-ventral axis. At *k* = 6, the putamen was divided into rostral and caudal clusters for left side, while was divided into rostrodorsal, rostroventral and caudal clusters for right side. In addition, for the left side, we found a dorsomedial cluster that derived from the dorsal caudate. At the highest level of subdivision (*k* = 7), the division of the putamen in the left side was similar to that of the right side at 6-cluster solution. For the right side, we found an additional small cluster that derived from caudal putamen.

### 3.3 Selection of cluster solutions

We then investigated how these functional parcellation solutions of the striatum from low to high levels of subdivision were supported by the data itself based on several cluster selection criteria. Fig. S4-S7 and Table S5 show the results of cluster solution criteria for both human and macaque striatum.

*Human striatum (Fig. S4-S5):* both the Hierarchy index and variation of information across clusters criteria suggested the 6-cluster solution over the others for left side. The Hierarchy index also supported the 3-cluster solution for both side, and 6-cluster solutions for right side.

*Macaque striatum (Fig. S6-S7):* both the percentage of misclassified voxels and silhouette value criteria supported the 6-cluster solution for both side. The Hierarchy index criterion suggested *k* = 3 for both side, while the variation of information criterion supported a 5-cluster solution for the left side, and 3-cluster solution for the right side.

Most criteria supported 3 and 6 cluster solutions which can be regarded as the stable solutions in both left and right striatum in both human and macaque CBP analysis. Thus, for subsequent analyses we focused on these parcels and compared their cortico-striatal RSFC across human and macaque.

### 3.4 Three and six parcels of human and macaque striatum

**Fig. 3:**
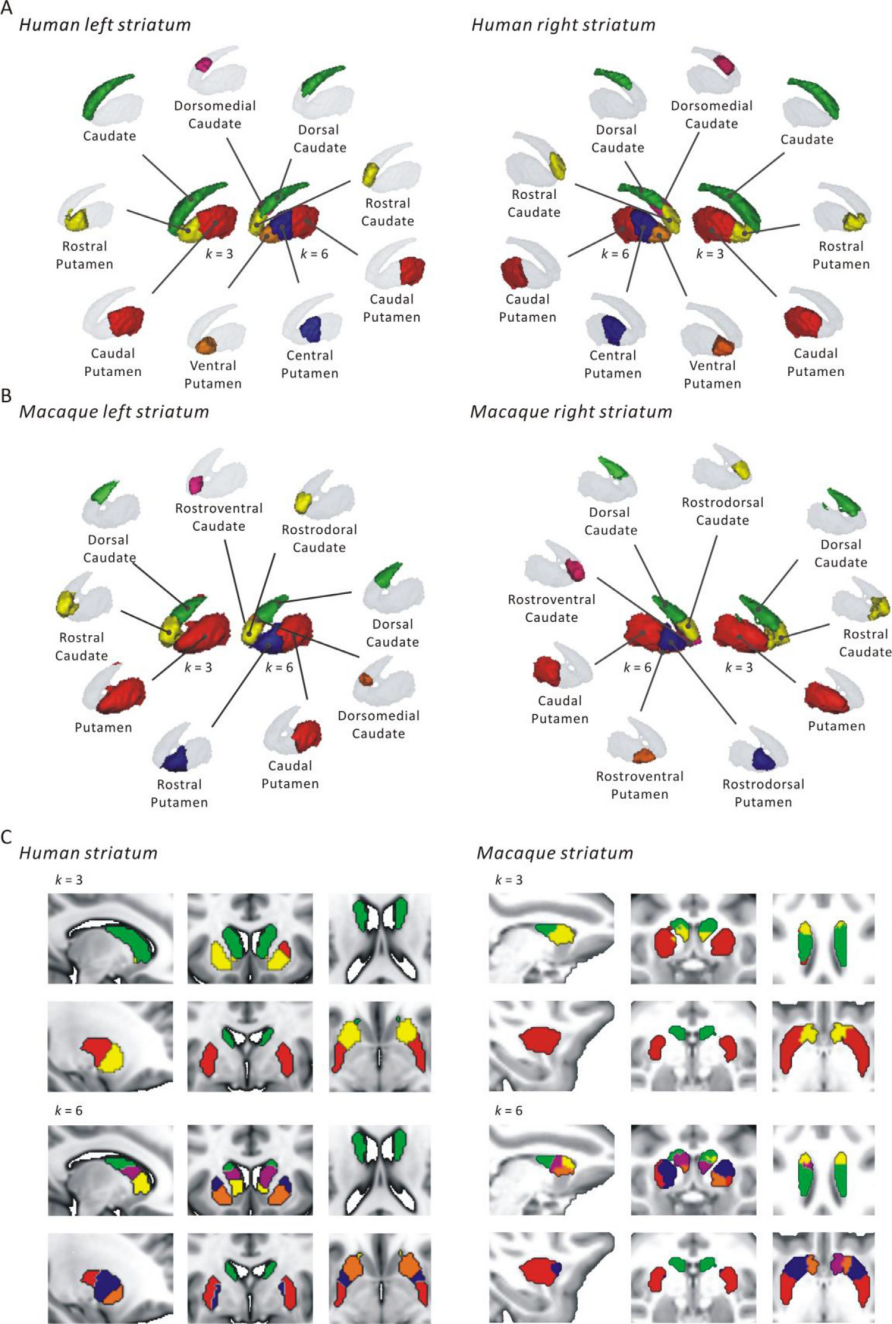
The location of each cluster for human (A) and macaque (B) striatum at 3 and 6 clusters solutions. The sagittal, coronal and cross section views (C) provide detailed localization of the clusters.

Fig. 3 shows detailed location information of human and macaque striatal parcels with 3 and 6 clusters. For *k* = 3, the human left and right striatum were subdivided into caudate, rostral and caudal putamen (Fig. 3A). In macaque, left and right striatum were split into putamen, rostral and dorsal caudate at this solution (Fig. 3B). For *k =* 6, the parcellation of the human left and right striatum were also similar, which include dorsal, dorsomedial and rostral caudate and ventral, central, and caudal putamen (Fig. 3A). In macaques, the parcellation of the left and right striatum were slightly different for the 6-cluster solution (Fig. 3B). We found dorsal, rostrodorsal, rostroventral caudate and caudal putamen for both left and right side. They differed only in that the left side had an additional dorsomedial caudate, while the right side had an additional rostroventral putamen. The details for the between subject and individual-level and group-level parcels are provided in the Supplementary Materials (Fig. S8).

### 3.5 CBP robustness checks

We performed several control analysis to check the robustness of our clustering solutions. First, we performed split-half analysis to check the effect of sample selection and found the group-level clustering solutions to be stable across 1000 random splits of the data. We then reanalyzed the HCP data but this time after applying the FIX-based denoising which provides an alternate way to remove motion artifacts. We found a high level of match using the adjusted rand index (ARI) between the reported parcels and the FIX-denoised parcels; 0.86 (*k* = 3) and 0.69 (*k* = 6) for the human left striatum and 0.88 (*k* = 3) and 0.75 (*k* = 6) for the human right striatum for the selected clustering solutions. We then performed two replication analyses. These results were highly similar with the solutions obtained in our main analysis [all adjusted rand index (ARI) above 0.58]. The second replication was performed on a separate HCP sample which showed all ARI above 0.56 (Table S4). To further validate our human striatum parcellation, we estimated the RSFC between the striatal clusters (at *k* = 6) and seven cortical networks (Yeo et al., 2011) based on averaged RSFC across all subjects. We observed that the RSFC patterns between our striatal clusters and the seven cortical networks was similar to that of Choi et al. (2012) (Fig. S10) showing that our RSFC based functional parcellation is in line with a previous large-scale study. Details of these control analyses are provided in the Supplementary Materials.

We also performed robustness checks for the macaque clustering solutions. Specifically, we performed split-half analysis, and compared the main solutions (*k* = 3 and 6) with that obtained using only anesthetized subjects (all ARI above 0.9) and with a hold-out sample of 12 subjects (all ARI above 0.3). Several additional tests were performed to check the validity of the human and macaque clustering solutions including a different way to obtain the group-level parcellation, permutation tests and removal of border voxels (see Supplementary materials and Table S6). We further tested the sensitivity of the group-level clustering to sample size and our results show that larger samples, as used here, provide a better signal resulting in more stable group-level parcellation (Fig. S9). These analyses indicated that our clustering solutions are robust (see Supplementary materials).

### 3.6 Cross-species comparison

**Fig. 4:**
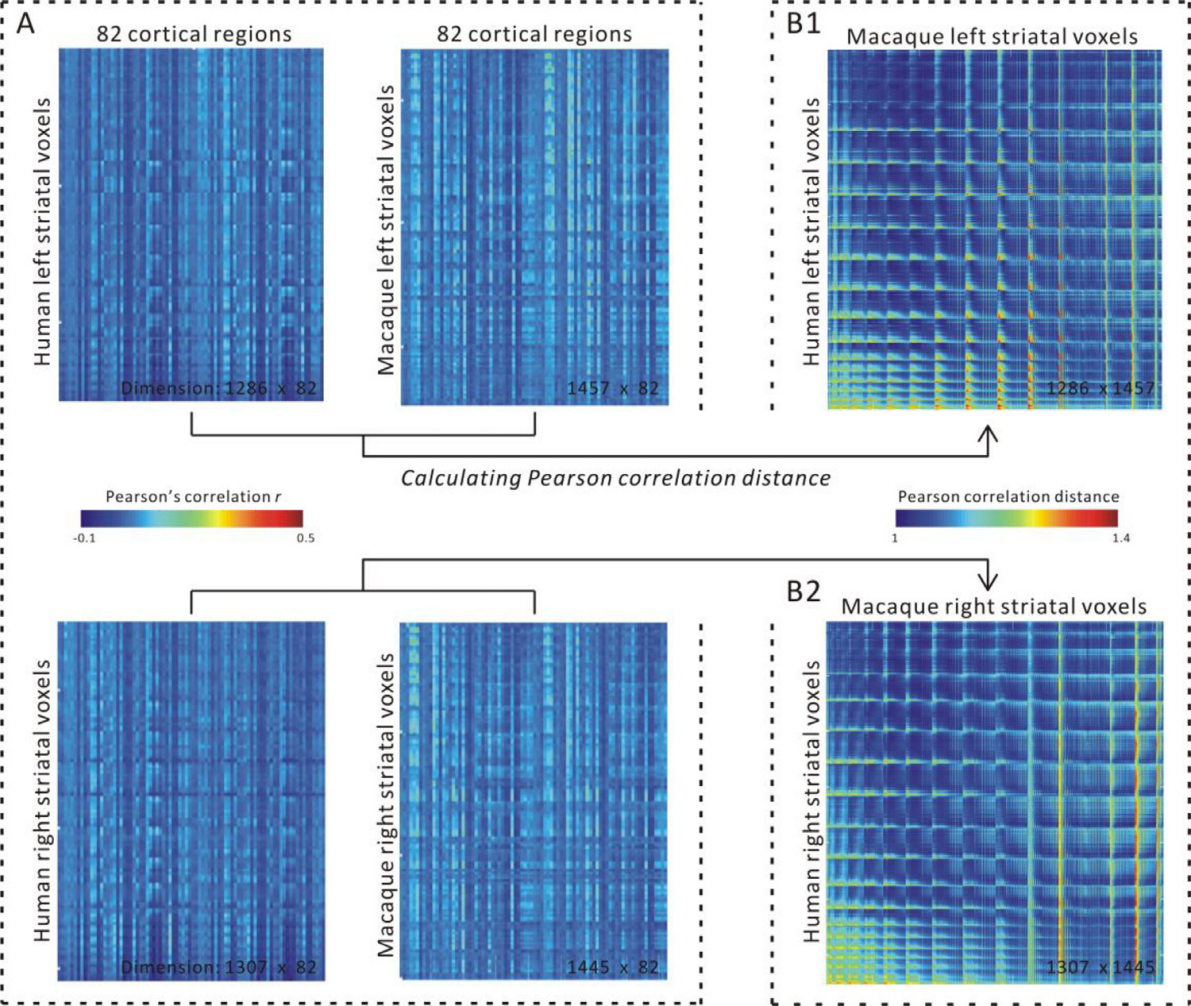
Cortico-striatal resting-state functional connectivity (RSFC) for human and macaque (A). Pearson correlation distance of each voxel based on cortico-striatal connectivity of human and macaque for cross-species comparison (B).

We used the regional map (RM)-derived 82 homologous cortical regions to calculate cortico-striatal RSFC comparable across human and macaque. Fig. 4A shows cortico-striatal RSFC between each left and right striatal voxel and cortical regions separately averaged across human or macaque subjects. We adopted the Pearson correlation distance metric to estimate the dissimilarity of connectivity between any given human and macaque striatal voxels (Fig. 4B1 left striatum, Fig. 4B2 right striatum).

#### 3.6.1 Cluster-based cross-species comparison

**Fig. 5:**
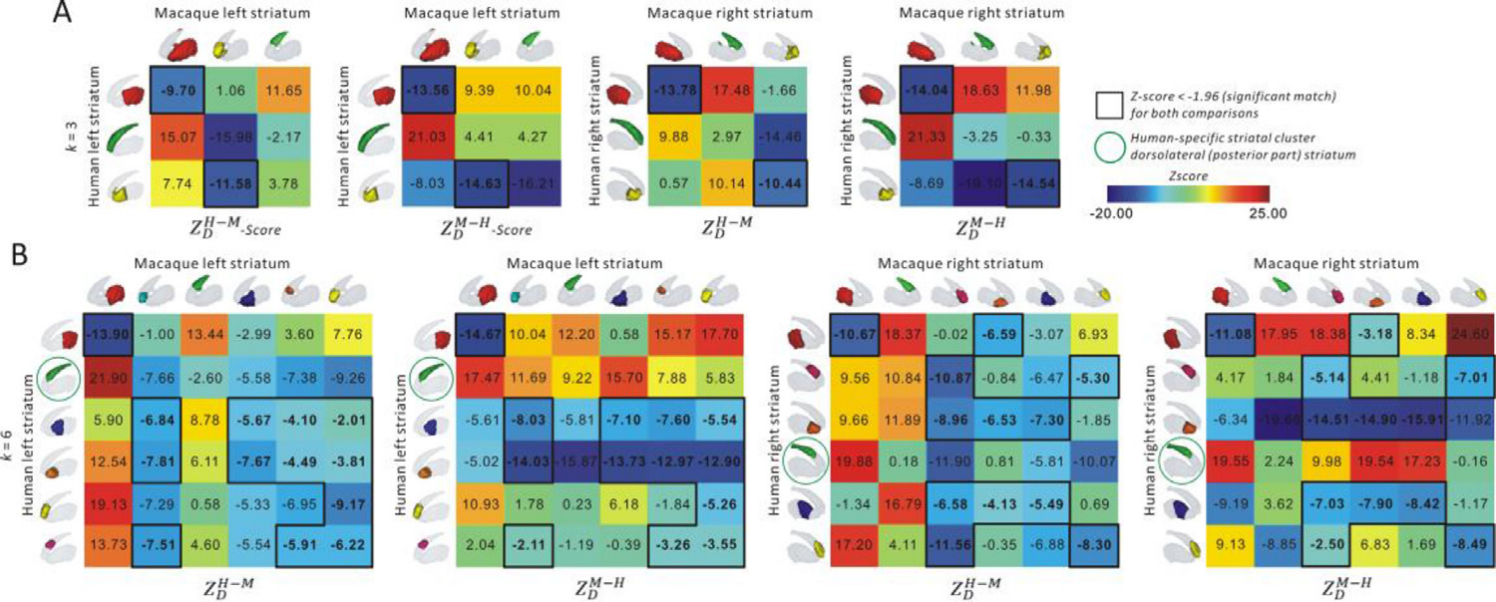
Permutation based *Z*-score of Pearson correlation distance for cluster-based cross-species comparison. The human and macaque clusters were generated from connectivity-based parcellation at *k* = 3 (A) and 6 (B).

We calculated permutation-based *Z*-scores to assess cross-species similarity of the functional parcels. Note that the we obtained two Z-scores, one after permuting macaque cluster assignments and the other after permuting human cluster assignments, and a human-macaque cluster pair was deemed to show significant similarity in their cortico-striatal RSFC only if both Z-scores were below the significance threshold (see Methods). We focused on the 3- and 6-cluster solutions as these were supported by various cluster selection criteria. For the 3-cluster solution, we found significantly similar cortico-striatal RSFC between human left and right caudal putamen and macaque left and right putamen, as well as between human left and right rostral putamen and macaque left and right rostral caudate (Fig. 5A). For the 6-cluster solution, significantly similar cortico-striatal RSFC was found between the human and macaque left caudal putamen (Fig. 5B). Meanwhile, the cortico-striatal RSFC of human left ventral and central putamen were significantly similar to that of macaque left rostral, dorsomedial caudate and rostral putamen. We also observed significant similarity in cortico-striatal RSFC between human left rostral caudate and macaque left rostrolateral caudate. For the right striatum, similar results were observed for human and macaque caudal putamen. The cortico-striatal RSFC of human right ventral and central putamen were significantly similar to that of macaque rostroventral caudate and rostral putamen. In addition, we also found the human right dorsomedial and rostral caudate have significant similar cortico-striatal RSFC with macaque right rostral caudate. In summary, we found similar cortico-striatal RSFC in dorsomedial, rostral caudate and putamen between humans and macaques at 3- and 6-cluster solutions.

Interestingly, we found that the dorsal caudate cluster in humans did not match with any of the macaque clusters, especially not with spatially similar macaque cluster. This cluster was therefore labeled as showing human-specific cortico-striatal RSFC.

#### 3.6.2 Cross-species difference in cortico-striatal RSFC of dorsal caudate

**Fig. 6:**
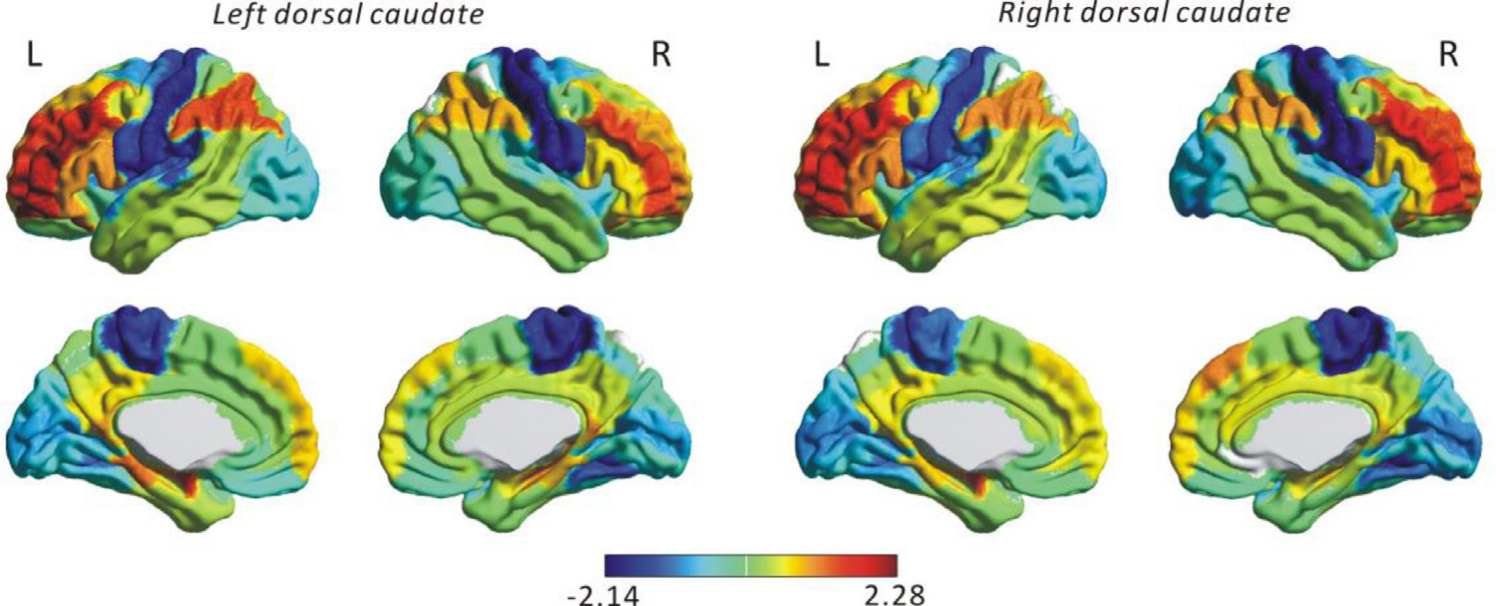
Difference in cortico-striatal resting-state functional connectivity (RSFC) of dorsal caudate (at *k* = 6) between human and macaque. The cortical regions with positive value represent stronger connection with the human dorsal caudate, while a negative value reflects stronger connectivity between macaque dorsal caudate and homologous cortical regions. The difference in RSFC values were Z-scored for visualization ease.

To further investigate the cortical connectivity of the human-specific parcel, we calculated difference in the connectivity of the human and macaque dorsal caudate parcels based on its connectivity with the corresponding homologous 82 cortical RM ROIs (Fig. 6). For both left and right side, we found the human dorsal caudate to be more strongly connected to prefrontal regions while the macaque dorsal caudate to be more strongly connected to the visual areas and to the pre/postcentral gyri. The pre/postcentral gyri are a part of the sensorimotor circuits, with the precentral gyrus mainly related to motor functions while the postcentral gyrus corresponds to the primary somatosensory cortex (Johns, 2014).

### 3.7 Difference in cortico-striatal RSFC of striatal clusters between patients and HC

**Fig. 7:**
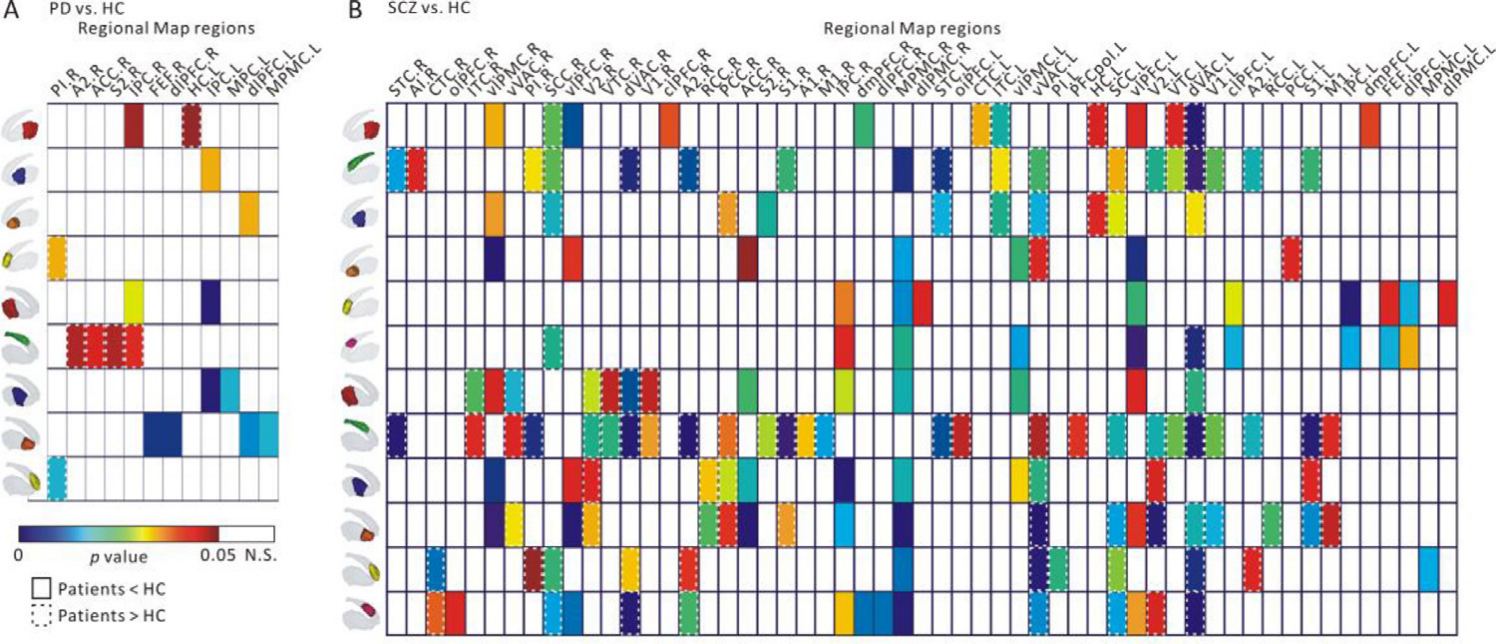
Significant differences in cortico-striatal RSFC of striatal clusters (at *k* = 6) between A) PD patients versus HC, B) SCZ patients versus HC. The color boxes represent significant *p* value (*p* < 0.05, FDR corrected). The solid boxes represent RSFC in HC > Patient, while the dashed boxes represent RSFC in Patient > HC. Abbreviation: PD, Parkinson's disease; SCZ, schizophrenia; HC, healthy controls; N.S., no significant difference.

We found significant differences in cortico-striatal RSFC of multiple striatal clusters for both PD and SCZ compared to HC (Fig. 7).

For PD, cortico-striatal RSFC of left and right ventral, central, caudal putamen and rostral caudate was significantly different than HC (*p* < 0.05, FDR corrected). Significantly weaker RSFC between central putamen and caudal putamen with inferior parietal cortex (IPC) were found in PD compared to HC. A significantly stronger RSFC in PD compared to HC was found between right dorsal caudate (i.e., the human-specific cluster) and IPC, anterior cingulate cortex (ACC), secondary somatosensory cortex (S2), and secondary auditory cortex (A2).

For SCZ, the human-specific dorsal caudate cluster showed the most significant differences between patients and controls on both left and right sides, 18 and 26 respectively (Fig. 7), with the majority of them higher in SCZ (17 and 26, respectively). For this cluster, SCZ showed a significantly higher RSFC with the temporal cortices including superior temporal cortex (STC and VTC), visual areas (V1 and V2, dVAC and ventral part vVAC), A2, primary somatosensory cortex (S1), and subgenual cingulate cortex (SSC). Significantly lower RSFC between caudal putamen and ventrolateral prefrontal cortex (vlPFC), ventrolateral premotor cortex (vlPMC), but a significantly higher RSFC between this striatal cluster and temporal cortices (inferior and ventral, ITC and VTC), and anterior visual area (dorsal part, dVAC) were found in SCZ compared to HC. Significantly lower RSFC between central putamen and vlPMC, but significantly stronger RSFC between this cluster and posterior cingulate cortex (PCC) and vVAC were found in SCZ than in HC. For ventral putamen, we found significantly lower RSFC in SCZ patients as compared to HC between this striatal cluster and vlPFC, vlPMC, medial premotor cortex (MPMC), and ACC. However, we also found significantly higher RSFC between this striatal cluster and PCC, and vVAC., We found significant differences between the connectivity of left side rostral caudate with prefrontal, parietal, and premotor cortices, while significant differences between connectivity of right rostral caudate with visual and auditory areas and central temporal cortex (CTC). In addition, although we found no significant difference in cortico-striatal RSFC of the dorsomedial caudate between PD and HC, some significant results were detected between SCZ and HC. For example, significantly lower RSFC between dorsomedial caudate and IPC, MPMC, and dorsolateral prefrontal cortex (dlPFC), while significantly higher RSFC between this striatal cluster and SSC and dVAC were found in SCZ compared to HC.

Taken together, we found significant difference in cortico-striatal RSFC of central, ventral putamen and rostral caudate between patients (PD and SCZ) and HC. Interestingly, for the dorsal caudate (i.e., the human-specific cluster), almost all significant results showed a higher cortico-striatal RSFC in patients (PD and SCZ) compared to HC. In addition, for SCZ, most of the significant differences were observed between this striatal cluster and visual areas, auditory and somatosensory cortex.

### 3.8 Alteration in GM volume of striatal clusters in PD and SCZ

We analyzed how “disease status”, “striatal clusters”, “age”, “sex”, “hemisphere” and “sites” are related to the average GM volume of the striatum by applying a six-way ANOVA.

**Main effect**

**Fig. 8:**
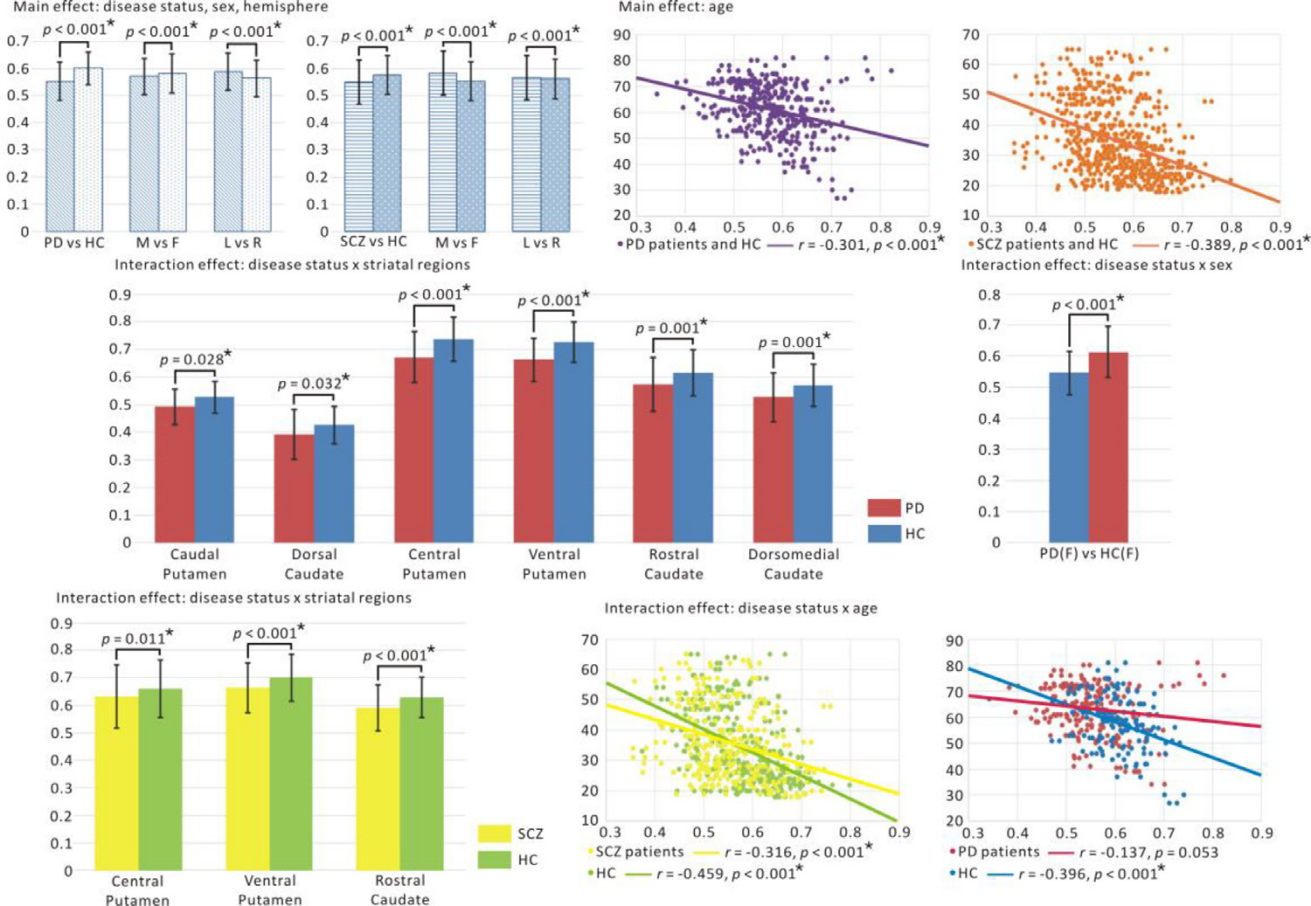
Difference in VBM of striatal clusters (at *k* = 6) between patients with (PD, SCZ) and HC. Abbreviation: PD, Parkinson's disease; SCZ, schizophrenia; HC, healthy controls; L(R), left (right) hemisphere; M (F): male (female).

We found significant main effects of “disease status”, “striatal clusters”, “sex”, “age”, “hemisphere” and “sites” on the average GM volume in both disorders (Fig. 8). Both PD and SCZ patients showed significantly lower GM striatal volume compared to HC (*p* < 0.001). When combining PD and HC, male subjects had a significant lower GM volume than female subjects, but we found an inverse result in SCZ and HC dataset. Younger subjects had a higher GM volume than older subjects. The correlation analysis showed a significant negative correlation between the GM volume of the striatum and age (PD and HC: *r* = −0.301, *p* < 0.001, SCZ and HC: *r* = −0.389, *p* < 0.001). In addition, we found significant higher GM striatal volume in left hemisphere compared to right hemisphere (*p* < 0.001).

**Interaction effects**

We then focused on the interaction effects of the factor “disease status” (PD and SCZ separately) with other factors (i.e., “striatal clusters”, “sex”, “age” and “hemisphere”) (Fig. 8). We found significant lower GM volume of all striatal clusters in PD compared to HC. In female subjects, PD patients have lower GM volume of the whole striatum than HC. Significant lower GM volume of central, ventral putamen and rostral caudate were found in SCZ compared to HC. No significant difference in GM volume of the human specific striatal cluster (dorsal caudate) was found between SCZ and HC. Moreover, we also found significant interactions between “disease status” and “age”, showing a significant negative correlation between GM volume of the striatum and age in both SCZ patients (*r* = −0.316, *p* < 0.001) and HC (*r* = −0.459, *p* < 0.001, Fig. 8).

## 4 Discussion

This study investigated functional parcellation of human and macaque striatum. To this end, we first demarcated the functional regions of human and macaque striatum using connectivity based parcellation (CBP) based on whole-brain RSFC. Three and six striatal cluster solutions for both human and macaque were selected based on various data-driven model selection criteria. We found a dorso-ventral and a rostro-caudal topographical organization in both human and macaque striatum. We then used these clusters as a basis to estimate cross-species similarity between pairs of human-macaque clusters based on their RSFC with 82 Regional Map (RM) homologous cortical regions. Significant similarity in the cortico-striatal RSFC were found between the human and macaque rostral caudate and putamen for both left and right side. However, there was no significant similarity in the cortico-striatal RSFC between human dorsal caudate and any of the macaque striatal clusters. Further analysis of this human-specific dorsal caudate cluster revealed cross-species differences in its cortico-striatal RSFC, especially with the prefrontal regions, somatosensory cortex and visual areas. When probing for clinical significance, RSFC between this human-specific cluster (dorsal caudate) with visual area, auditory cortex, IPC, somatosensory cortex were found to be stronger in SCZ than HC. Also, structural atrophy in this human-specific cluster was found in PD patients compared to HC.

### 4.1 Functional parcellation of the human striatum

The human striatum was divided into caudate and putamen at the simplest parcellation with *k* = 2. Although informative as a baseline, this solution was not selected by our data-driven model selection suggesting a more complex functional organization of the striatum. Solutions with 3 and 6 clusters were selected by various selection criteria (Table S5) and are discussed below in detail.

At the 3-cluster solution for the human striatum, we observed a caudate nucleus cluster and the putamen was split into rostral and caudal parts (Fig. 3). Our parcellation of the putamen at this solution is similar to recent study that parcel the putamen into anterior and posterior part based on functional connectivity gradients (Tian et al., 2020). Finding different parcels associated with the caudate nucleus and the putamen is in line with the modular view of the striatum and the different functions of these two major modules (Grahn et al., 2008). Although the traditional view suggests that the putamen is more associated with motor functions, several studies have demonstrated that it is also related to various cognitive processes including learning and memory (Ell et al., 2011; O'Doherty et al., 2004). Choi et al. (2012) investigated RSFC between striatal subregions and seven cortical networks (visual, somatomotor, dorsal attention, ventral attention, limbic, frontoparietal and default mode) as identified by Yeo et al. (2011). They found that the ventral attention and frontoparietal networks were connected with the rostral putamen, while the somatomotor network was primarily connected with the caudal putamen. In addition, Jung et al. (2014) found the rostral putamen was positively linked to affective and cognitive control cortical regions, whereas the caudal putamen was positively linked to more motor control cortical regions. Pauli et al. (2016) analyzed 5809 functional imaging studies and estimated task-based functional co-activation patterns of the striatal voxels with the cerebral cortex. According to these co-activation patterns, the putamen was divided into the rostral part that was related to social and language functions, and the caudal part associated to sensorimotor processes. In line with these previous findings our functional parcellation of the putamen suggests a differentiation in intrinsic RSFC and functions between its rostral and caudal parts.

The 6-cluster results were generally similar across hemispheres and showed an overall similarity with our previous multi-modal CBP of the human striatum (Liu et al., 2020). The RSFC patterns between our striatal clusters and the seven Yeo cortical networks was similar to that of Choi et al. (2012) (Fig. S10). Similar parcellation were shown in the Garcia-Garcia et al. (2018) study which found stable striatal clusters including the rostral, dorsal caudate, caudal putamen across three scans. Another study (Kim et al., 2013) applied the temporal independent component analysis (ICA) to cluster the basal ganglia and the thalamus into 31 functional subdivisions. They found the caudate was divided into head, body and tail parts. In our parcellation, the rostral part of the caudate was close to the head, while the dorsal and dorsomedial parts approximately matched the body and the tail of the caudate, respectively. Differences in functional connectivity of meta-analytic connectivity modeling (MACM) between the head and body/tail of the caudate nucleus revealed that the head of the caudate is more involved in cognitive and emotional processes compared to the body and tail (Robinson et al., 2012). In addition, our striatal clusters were similar to that in Janssen et al. (2015) study, who found the caudate was divided into dorsal, ventral, rostral part while the putamen was split into dorsal, rostral, caudal part. The slight difference is likely caused by differences in data and clustering methods.

Our functional parcels of human striatum were partly in agreement with previous structural CBP of the striatum. Leh et al. (2007) applied DTI tractography to examine the structural connectivity between the frontal cortex and the striatum. They found the dlPFC projects to dorso-caudal caudate while vlPFC projects to ventro-rostral caudate. For putamen, structural connectivity was found between supplementary motor area (SMA) and dorso-caudal putamen, between premotor area and medial putamen, as well as between primary motor area lateral putamen. In another study, Tziortzi et al. (2014) used similar methods and showed that the frontal lobe projects to almost the whole caudate, the rostral and central putamen while the parietal lobe projects to caudal caudate and dorso-caudal putamen. Small-interspersed projections from the temporal and occipital lobe were observed in the ventro-caudal putamen. Overall, these structural connectivity based studies divide the striatum along the dorso-ventral and rostro-caudal axes. Similar to these findings, our functional parcellation at 6-cluster solution discerned rostral, dorsal caudate and ventral, caudal putamen, which may suggest a convergent functional and structural organization of these striatal clusters (Liu et al., 2020).

### 4.2 Functional parcellation of the macaque monkey striatum

Data-driven model criteria also suggested 3 and 6 cluster solutions for the macaque striatum, which are discussed here. We found the macaque striatum to be divided into rostral, dorsal caudate and putamen in the 3-cluster solution (Fig. 3). A previous study found the focal projections from lateral Brodmann Area 9 (BA9), a part of the frontal cortex, terminated in the dorsal caudate, while projections from BA46 terminated in the putamen (Calzavara et al., 2007). Previous studies (Johnson et al., 1968; Kemp and Powell, 1970) investigated the fiber degeneration and revealed a rostro-caudal organization for cortical terminal in striatal regions. Our parcellation of macaque striatum is generally consistent with these reports. The rostral caudate probably includes the major part of the head of the caudate. This region is assigned to the cortico-striatal loop that receives afferent projections from dorsolateral prefrontal cortex and lateral orbital frontal cortex, which is related to emotions, motivation and higher cognitive processes (Alexander et al., 1986; Haber, 2003). Our parcellation result confirms this differential RSFC. The parcellation results differed between human and macaque striatum at this clustering granularity. In humans, the putamen was split into rostral and caudal parts, while in macaques, the caudate was divided into rostral and dorsal parts. However, it should be noted that the information that can be gained from a low granularity parcellation is limited, given the complex functional organization of the striatum. We hence compared human and macaque striatum parcellation at the higher granularity of 6 clusters.

At the higher granularity of 6 clusters, the macaque rostral caudate from the 3-cluster solution was further divided into dorsal and ventral parts (Fig. 3). Previous non-human primate studies has shown that the dorsolateral part of the caudate head connects with the dorsolateral prefrontal regions, while the ventromedial part connects with the orbital frontal regions (Alexander et al., 1986; Goldman and Nauta, 1977; Künzle, 1978; Selemon and Goldman-Rakic, 1985; Yeterian and Van Hoesen, 1978). The dorsolateral part of the caudate head also receives projections from the arcuate premotor area and posterior parietal cortex (BA 7) (Künzle, 1978; Selemon and Goldman-Rakic, 1985). This striatal region is related to gold directed actions, such as working memory (Bonelli and Cummings, 2007). Our results are partly in line with and extend these previous findings, suggesting a convergent functional and structural connectivity of the rostral caudate/head caudate. In addition, compared with the parcellation of the human striatum at this 6-cluster solution, the human rostral caudate that was not split. This suggests a more homogenous RSFC within the human rostral caudate than within macaque rostral caudate.

Taken together, based on the CBP analyses we found differential functional parcellation of human and macaque striatum based on their RSFC, especially concerning the rostral caudate. These results may be related to disproportionate volumetric differences in the regions functionally connected with the striatum, such as human prefrontal cortex (Carlén, 2017; Smaers et al., 2017), hippocampus and amygdala (Barger et al., 2014) during primate evolution, and may have altered RSFC of these striatal regions and generated differential functional parcellations in humans and macaques.

### 4.3 Cross-species comparison based on homologous cortico-striatal RSFC

We compared human and macaque striatum parcellation results in a data-driven fashion, which allowed us to quantify and localize the extent of similarity and differences.

Similarity in the cortico-striatal RSFC of the rostral caudate and putamen was observed between human and macaque (Fig. 5). Although correspondence between RSFC and microstructural connectivity features of subcortical regions remains poorly understood (Moerel et al., 2014; van den Heuvel et al., 2015), our findings supplement previous studies showing similarities in the cellular and molecular composition and distribution, as well as functions of the striatum across species (Betarbet et al., 1997; Haber and Knutson, 2010; Hardman et al., 2002; Lohrenz et al., 2016). For example, the caudal putamen is likely more related to motor functions in humans (Marchand et al., 2008). Similarity in cortico-striatal RSFC of the corresponding cluster between human and macaque suggests a similar mechanism and ability of primary action execution. This result is in line with recent findings that functional involvement of caudal/lateral putamen in cortico-striatal motor circuits are similar across human, macaque and mouse (Balsters et al., 2020). Both humans and monkeys adopt saccadic eye movements to search for objects in a crowded scene (Henderson, 2003; Sheinberg and Logothetis, 2001). The saccade patterns may change with different goals and thoughts (Yarbus, 2013). The caudate in macaque monkeys participates in the visual selection based on the value of the visual objects (Hikosaka et al., 2014). Our finding of significant cross-species similarity of cortico-striatal RSFC of the rostral caudate may reflect somewhat comparable mechanism relating to reward value-based selection behavior. In addition, several striatal clusters (e.g., rostrodorsal and rostroventral caudate) and their cortico-striatal RSFC were similar between human and macaque albeit not highly similar based on permutation *Z*-scores (Fig. 5B). Perhaps this is due to the similar connectivity and function of these adjacent striatal clusters. Hence, similar cortico-striatal RSFC of human rostral caudate with macaque rostral caudate, as well as with macaque rostral putamen could be detected, given striatal clusters in these locations are related to emotion and cognitive functions (Liu et al., 2020).

Intriguingly, there was no significant similarity in the cortico-striatal RSFC of the topographically similar dorsal caudate in the two species (Fig. 5B). This may suggest a functional modification of the dorsal caudate during evolution. The caudate nucleus plays an important role in integrating visual information and reward context in decision-making (Doi et al., 2020). The dorsal caudate connects with dlPFC (Choi et al., 2012; Robinson et al., 2012), and this circuit is associated with generation of motivation, including the expected reward of action, and prediction of action-outcome contingency (Balleine et al., 2007; Haber and Knutson, 2010; Mucci et al., 2015). In humans, the activation of dorsal caudate has been observed during anticipation of reward, which was related to real-life motivation (Mucci et al., 2015). Based on the expected reward, the dorsal caudate may mediate action selection as well as associating these actions with outcome in goal-directed behaviors. Accumulated evidences (Balleine et al., 2007; Burton et al., 2015; Hiebert et al., 2017; Hollerman et al., 1998) suggest that the striatum is a crucial part of a circuit related to reward and decision-making in both human and non-human primates. However, the caudate plays a different role than the putamen as discussed above and whether involvement of the dorsal caudate in goal-directed behaviors is affected by various complexities in human social interactions and induce alteration in its functional and structural connectivity between human and non-human primate is still not known. During primate evolution, humans have presumably encountered different rewards, decision-making and social communication tasks than non-human primate (Santos and Rosati, 2015), which may have induced differential neural activity of the dorsal caudate reflected in the presently observed dissimilar cortico-striatal RSFC between human and macaque. In a previous study (Neubert et al., 2015), differential functional and structural connectivity between cortical regions relating to reward-guided learning and decision-making between human and macaque have been reported. Our findings showed dissimilar cortico-striatal RSFC of dorsal caudate relating to relevant reward and decision-making functions can be detected in cross-species comparison supplementing previous studies. In addition, further post-hoc analysis revealed the macaque dorsal caudate to be strongly connected to somatosensory cortex (pre/postcentral gyri) and visual areas, while this region in human was more strongly connected to prefrontal regions (Fig. 6). The pre/postcentral gyri are related to primary motor and sensory functions, while the prefrontal regions are involved emotion, complex cognitive control and motor functions. This difference in cortico-striatal RSFC of the dorsal caudate may suggest its more involvement in relevant reward and goal-directed behaviors in humans compared to macaques. A recent study in humans showed that de novo motor skill learning induces anterior-to-posterior transition of fMRI activity in the caudate nucleus and suggested that stronger visual-caudate tail functional connectivity, as we found in macaques, hinders skillful performance (Choi et al., 2020). In this context, our dorsal caudate cluster which partly overlaps with the caudate tail suggests that this potentially evolutionarily divergent cluster is essential for learning complex skills and might set us apart from non-human primates.

In sum, similarity between humans and macaques in the cortico-striatal RSFC of the striatal clusters may reflect a functional homology of rostral caudate and the whole putamen. On the other hand, differences in cortico-striatal RSFC of the dorsal caudate may be due to differences in evolutionary pressure (e.g. due to type of social interactions, and the need to acquire complex skills). We speculate that the RSFC of the dorsal caudate may have evolved to become more strongly connected to the frontal regions, in effect increasing its involvement in more complex social functions. This complements, interspecies differences in brain structure (e.g., size and gyrification) and structural connectivity (Heuer et al., 2019; Krubitzer and Kaas, 2005).

### 4.4 Clinical relevance of cortico-striatal RSFC and structural alteration in striatal clusters

We first investigated alteration in cortico-striatal RSFC of our striatal clusters between patients (PD and SCZ) and HC.

The dysfunction of cortico-striatal circuitry has been implicated in psychiatric and neurological diseases. Stephens et al. (2005) showed reduced density of dendritic spines in both caudate and putamen by analyzing postmortem tissue in PD patients. This indicates a breakdown of cortico-striatal connections in PD as dendritic spines receive crucial excitatory input from the cerebral cortex. Previous in-vivo neuroimaging studies have revealed abnormal cortico-striatal RSFC in PD (Helmich et al., 2010; Hou et al., 2016; Luo et al., 2014). Helmich et al. (2010) found PD patients to show significantly decreased RSFC between caudal putamen and the inferior parietal cortex (lPC) while increased RSFC between rostral putamen and IPC. The caudal putamen is associated with motor functions in cortico-striatal circuitry. Hyperactivity of lPC has been observed during simple finger movement, which may suggest that PD patients may highly recruit this sensorimotor region during simple motor tasks (Samuel et al., 1997). Decreased cortico-striatal RSFC between caudal putamen and IPC supports commonly observed dysfunction of motor networks in PD patients. Our results are in line with those previous findings. In addition, previous nuclear imaging and postmortem studies (Brück et al., 2006; Guttman et al., 1997; Kish et al., 1988) have showed that the severity of dopamine depletion in caudal putamen is relatively higher than other striatal clusters in PD patients. We also found increased RSFC between dorsal caudate (i.e., the human-specific cluster) and the IPC. Given dopamine depletion of the caudate is relatively slight in PD patients, this finding may reflect a compensatory mechanism. That is, increased RSFC between dorsal caudate and IPC may compensate for severe dopamine depletion of caudal putamen inducing decreased coupling with IPC.

The dysfunction of the striatum is considered fundamental in different hypotheses of the etiology of SCZ (Fatemi and Folsom, 2009; Howes and Kapur, 2009). Dopamine dysregulation in the striatum have been found in SCZ, and it has become the primary target for several antipsychotic drugs (Kapur, 2003). Previous neuroimaging studies have reported abnormal RSFC in cortico-striatal circuits in SCZ. In the current study, significantly stronger cortico-striatal RSFC in SCZ was found for all striatal clusters with at least one of the cortical RM ROIs (Fig. 7). This result was in line with previous studies that showed increased RSFC between striatum and various cortices, including prefrontal, temporal and cingulate cortex, in SCZ patients (Kirino et al., 2019; Salvador et al., 2010). This increased cortico-striatal RSFC may reflect disruption of segregation between subcortical and cortical functional network (Kirino et al., 2019). Specifically, we found significantly stronger RSFC between striatal clusters (dorsal caudate, ventral and central putamen) and posterior cingulate cortex (PCC) which is a part of the default mode network (DMN). Another previous study (Salvador et al., 2010) reported hyper-connectivity between caudate and medial orbital prefrontal cortex—another cortical region within the DMN—suggesting an overall disruption of DMN-striatum relationship in SCZ. Our findings support this viewpoint as we found hyper-connectivity between more widespread regions within the DMN-striatum loop—ventral and central putamen and PCC. We also found significantly stronger RSFC in SCZ between dorsal caudate (human-specific cluster) and widespread cortical regions, including temporal, visual areas, secondary auditory cortex and primary somatosensory cortex. Interestingly, macaques showed a stronger connectivity of an anatomically similar cluster with visual and somatosensory areas than healthy humans (Fig. 6). The dopaminergic innervation of dorsal caudate is elevated in humans compared to macaques (Raghanti et al., 2016). This increased dopaminergic input may reflect functional connectivity between cortical regions to dorsal caudate and involve specific behavioral and cognitive functions in humans like speech production and language whose disturbance is a core symptom of schizophrenia (de Boer et al., 2020). Hence, the dopamine depletion of dorsal caudate may induce relevant cognitive impairments in SCZ patients. Combined with these reports, our finding suggest that human-specific reorganization in cortico-striatal RSFC between dorsal caudate and diverse cortical regions may play a vital role in the pathophysiology of SCZ. Taken together, this may suggest stronger involvement of dorsal caudate in more complex reward and decision-making and goal-directed behaviors in humans than in macaques, and consequently its impairment may be associated to abnormal cognition and behavior symptomatic to SCZ patients.

We also observed significantly lower GM volume of all striatal clusters for PD, and significantly lower GM volume of rostral caudate, ventral and central putamen for SCZ patients compared to HC (Fig. 8). Structural atrophy of striatum in PD and SCZ patients have been reported (Gaser et al., 2004; Hanganu et al., 2014; Lewis et al., 2016), which suggests that dopamine depletion induces morphological changes in the striatum. Our results provide a potential structural correlate within striatal clusters mirroring the dopaminergic dysfunction of striatum in PD and SCZ (McCutcheon et al., 2019). While most previous studies (du Plessis et al., 2018; Juckel et al., 2006; Rodriguez-Oroz et al., 2009) examined the functional and structural alterations of the ventral striatum, we could show that central putamen could be similarly relevant when investigating PD and SCZ pathology. We also found different alterations in RSFC and GM volume of striatal clusters, for example, no structural alterations of dorsal and dorsomedial caudate, and caudal putamen, while significant differences were observed in RSFC of these striatal clusters with cortical regions in SCZ. Also, the altered RSFC and GM volume of striatal clusters are not in full accord between PD and SCZ, suggesting differential pathophysiology of these diseases. Despite similar pathophysiology between PD and SCZ, i.e. abnormal depletion of striatal dopamine inducing functional and structural alteration of striatal clusters, their physiological mechanisms need further investigation.

### 4.5 Limitations and future work

We acknowledge several limitations of the present study which can be addressed in the future when more data becomes available and aided by development of novel tools [In this Issue, Xu (2021)]. First, the RM atlas was used as homologous cortical regions between human and macaque. We acknowledge that this atlas may not provide a perfect homology between neocortical areas of humans and macaques. Yet, based on cytoarchitectonic, gross anatomical, and functional criteria, minimizing cross-species discrepancies in ontology (Kötter and Wanke, 2005; Reid et al., 2016), the RM seems currently the most appropriate atlas available to compare cortico-striatal connectivity between humans and macaques. With new methods for cross-species comparison being developed (Xu et al., 2020b), adapting them for regional and parcel-based comparison is an interesting methodological question which, if addressed, can further increase the specificity and sensitivity of cross-species comparison analyses. Future studies using alternative approaches to defining homologous cortical atlas, or comparison of cortico-striatal RSFC in the cross-species common space may provide further insights.

Second, we used the *k*-means clustering algorithm to divide the human and macaque striatum voxels into specified number of *k* non-overlapping clusters based on the RSFC pattern of each striatal voxel. The *k*-means clustering algorithm has been successfully employed in several CBP studies investigating different brain regions and modalities (Chase et al., 2020; Crippa et al., 2011; Genon et al., 2018; Hartwigsen et al., 2019; Jung et al., 2014; Pauli et al., 2016; Plachti et al., 2019b; Ray et al., 2015; Reuter et al., 2020; Xu et al., 2020a) and is known to provide highly accurate solutions (Thirion et al., 2014). However, it is also known that *k*-means algorithm can be unstable and provide sub-optimal solutions. To circumvent this issue, in our analyses each *k*-means clustering was performed with 100 different initializations.

Third, following suggestions during the review process, we attempted to improve the alignment between individual-level and group-level clusters by removing edges from the connectivity matrix at different thresholds (5%, 25%, and 50%). The results are shown in Fig. S11 and Table S7. We obtained similar but slightly lower ARI values when comparing individual-level and group-level clusters. Future studies should focus on improving the alignment between individual-level and group-level using other CBP pipelines, e.g. using different clustering algorithms like spectral clustering (Arslan et al., 2018), employing bagging (Nikolaidis et al., 2020), and incorporating spatial constraints (Craddock et al., 2012; Schaefer et al., 2018).

Fourth, our aim was to identify striatal clusters in humans whose RSFC with cortical regions differs from macaques. This human-specific RSFC, if evolutionary, can be either adaptive (protects from a disease) or mal-adaptive (promotes a disease). Although based on our results in SCZ we speculate that the connectivity of this cluster might be mal-adaptive, our current analysis is not sufficient to either confirm or refute this hypothesis. Additional data from other primates and more disorders is needed to make informed claims.

A recent study found a trade-off between efficiency and robustness of neuronal activity in amygdala and cingulate gyrus (Pryluk et al., 2019), which might explain complexity of human behaviors and cognition compared to macaques, and why humans suffer from psychiatric diseases. It will be interesting to investigate whether such trade-offs exist in the RSFC of the human dorsal caudate, and probe if it explains disorders like PD and SCZ.

## Conclusions

Functional parcellation revealed that the human striatum was split into dorsal, dorsomedial, rostral caudate and ventral, central, caudal putamen, while the macaque striatum was divided into dorsal, rostral caudate and rostral, caudal putamen. The dorsal caudate showed dissimilar cortico-striatal RSFC between humans and macaques, suggesting its connectivity to be human-specific. Also, abnormal RSFC of this striatal cluster (among other clusters) with cortical regions was found in both PD and SCZ, while structural atrophy within this striatal cluster was observed only in PD. Taken together, our cross-species comparative results revealed shared and human-specific RSFC of striatal clusters reinforcing the complex organization and function of the striatum. Our investigations of whole-brain RSFC based parcellation and comparison of human and macaque striatum show that the RSFC may be used to compare functional organization between human and non-human primates. In addition to adding to our understanding of how human RSFC differs from that of macaque, our results also provide a testable hypothesis that abnormalities in a region with human-specific connectivity might be associated with complex neuropsychiatric disorders.

## Declaration of Competing Interest

All authors claim that there are no conflicts of interest.

## Appendix Supplementary materials

Image, application 1
