# Supplementary material for: Functional parcellation of human and macaque striatum reveals human-specific connectivity in the dorsal caudate
Source: Neuroimage. 2021 Jul 15;235:118006. doi: 10.1016/j.neuroimage.2021.118006 (PMC8214073; doi:10.1016/j.neuroimage.2021.118006)
Supplement: Supplementary file 1 [file mmc1.docx]

**Supplementary Materials**

**Functional parcellation of human and macaque striatum reveals human-specific connectivity in the dorsal caudate**

Xiaojin Liu^1, 2^, Simon B. Eickhoff^1, 2^, Svenja Caspers^3, 4^, Jianxiao Wu^1, 2^, Sarah Genon^1, 2^,

Felix Hoffstaedter^1, 2^, Rogier B. Mars^5, 6^, Iris E. Sommer^7^, Claudia R. Eickhoff^3, 8^, Ji Chen^1, 2^,

Renaud Jardri^9^, Kathrin Reetz^10, 11^, Imis Dogan^10, 11^, André Aleman^12^, Lydia Kogler^13^,

Oliver Gruber^14^, Julian Caspers^3, 15^, Christian Mathys^, 15, 16, 17^, and Kaustubh R. Patil^1, 2^*

^1^Institute of Systems Neuroscience, Heinrich Heine University Düsseldorf, Düsseldorf, Germany

^2^Institute of Neuroscience and Medicine (INM-7, Brain and Behaviour), Research Centre Jülich, Jülich, Germany

^3^Institute of Neuroscience and Medicine (INM-1), Research Centre Jülich, Jülich, Germany

^4^Institute for Anatomy I, Medical Faculty, Heinrich Heine University Düsseldorf, Düsseldorf, Germany

^5^Wellcome Centre for Integrative Neuroimaging, Centre for Functional MRI of the Brain (FMRIB), Nuffield Department of Clinical Neurosciences, John Radcliffe Hospital, University of Oxford, Oxford, United Kingdom

^6^Donders Institute for Brain, Cognition and Behaviour, Radboud University Nijmegen, Nijmegen, Netherlands

^7^Department of Biomedical Sciences of Cells & Systems, University Medical Center Groningen, Groningen, Netherlands

^8^Institute of Clinical Neuroscience and Medical Psychology, Medical Faculty, University of Düsseldorf, Düsseldorf, Germany

^9^Division of Psychiatry, University of Lille, CNRS UMR9193, SCALab & CHU Lille, Fontan Hospital, CURE platform, Lille, France

^10^JARA-BRAIN Institute Molecular Neuroscience and Neuroimaging, Forschungszentrum Jülich, RWTH Aachen University, Aachen, Germany

^11^Department of Neurology, RWTH Aachen University, Aachen, Germany

^12^Department of Neuroscience, University Medical Center Groningen, University of Groningen, Groningen, Netherlands

^13^Department of Psychiatry and Psychotherapy, Medical School, University of Tübingen, Germany

^14^Section for Experimental Psychopathology and Neuroimaging, Department of General Psychiatry, Heidelberg University, Germany

^15^Department of Diagnostic and Interventional Radiology, Medical Faculty, University of Düsseldorf, Düsseldorf, Germany

^16^Research Center Neurosensory Science, Carl von Ossietzky Universität Oldenburg, Oldenburg, Germany

^17^Institute of Radiology and Neuroradiology, Evangelisches Krankenhaus, University of Oldenburg, Oldenburg, Germany

* Correspondence should be addressed to

Kaustubh R. Patil, Ph.D.

Institute for Systems Neuroscience

Heinrich-Heine University Düsseldorf

and

Institute of Neuroscience and Medicine (INM-7)

Forschungszentrum Jülich

Jülich 52428, Germany

Email: k.patil@fz-juelich.de

**1. Data quality control**

The MRI Quality Control tool (MRIQC) (Esteban et al. 2017) was utilized to quantitatively and visually assess the data quality. Based on the raw data, this tool computes both structural and functional Image Quality Metrics (IQMs), e.g. signal-to-noise ratio (SNR) and contrast-to-noise ratio (CNR) (Magnotta et al. 2006) for anatomical scans, DVARS and framewise displacement (FD) for functional scans (Power et al. 2012), etc. Additionally, the tool generates a group report summarizing metrics for all subjects and individual subject reports; we utilize the individual reports for visual assessment of the T1-weighted image or the mean EPI image.

In the end, 7 macaque subjects were excluded, as strong artifacts affecting meaningful brain signal extraction were observed in these subjects. In 3 subjects, strong non-brain signals (possibly susceptibility artifacts) overlapped with brain signals. Nyquist ghost artifacts overlapping with brain signals were observed, to various extents, in all 7 subjects. Finally, signal loss in the cerebellum was observed in some subjects. Due to the location and small size and of the signal losses, we decided to include these subjects nonetheless.

**2. Data acquisition**

**Table S1.** Resting-state functional magnetic resonance imaging parameters

| Dataset | State | Scanner | TR (ms) | TE (ms) | FA | Number of slices | Voxel resolution (mm^3^) |
| --- | --- | --- | --- | --- | --- | --- | --- |
| *HCP (Human) dataset* | Awake | Siemens Skyra 3T | 720 | 33 | 52 | 72 | 2.0 x 2.0 x 2.0 |
| *PD dataset* | | | | | | | |
| Heinrich Heine University Düsseldorf | Awake | 3T | 2,200 | 30 | 90 |  | 3.1 x 3.1 x 3.1 |
| RWTH Aachen University | Awake | 3T | 2,200 | 30 | 90 |  | 3.1 x 3.1 x 3.1 |
| *SCZ dataset* | | | | | | | |
| RWTH Aachen University | Awake | Siemens TIM Trio 3T | 2,000 | 28 | 77 | 34 | 3.6 x 3.6 x 3.3 |
| Center for Biomedical Research Excellence | Awake | Siemens Trio 3T | 2,000 | 29 | 75 | 33 | 3.75 x 3.75 x 4.55 |
| University of Groningen | Awake | Philips Achieva3T | 2,400 | 28 | 85 | 43 | 3.44 x 3.44 x 3.00 |
| University of Göttingen | Awake | Siemens TIM Trio 3T | 2,000 | 30 | 70 | 33 | 3.0 x 3.0 x 3.0 |
| University of Lille | Awake | Philips Achieva 3T | 1,000 | 9.6 | 9 | 45 | 3.22 x 3.22 x 3.4 |
| Utrecht University | Awake | Philips Achieva 3T | 608 | 32.4 | 10 | 40 | 4.0 x 4.0 x 4.0 |
| *PRIME-DE project (Macaque) dataset* | | | | | | | |
| University of Oxford | Anesthetized | Undisclosed 3T | 2,000 | 19 | 90 | 35 | 2.0 x 2.0 x 2.0 |
| Institute of Neuroscience, China | Anesthetized | Siemens TIM Trio 3T | 2,000 | 29 | 77 | 63 | 1.5 x 1.5 x 1.5 |
| Newcastle University | Awake | Bruker Vertical 4.7T | 1,000 | 17 | 90 | 47 | 1.2 x 1.2 x 1.2 |
| University of California, Davis | Anesthetized | Siemens Skyra 3T | 1,600 | 24 | N/A | 35 | 1.4 x 1.4 x 1.4 |

Note: TR, repetition time; TE, echo time; FA, flip angle; N/A, not available; SCZ, Schizophrenia; PD, Parkinson’s disease.

**Table S2.** T1-weighted magnetic resonance imaging parameters

| Clinical data | Scanner | TR (ms) | TE (ms) | FA | Number of slices | FA | Voxel resolution (mm^3^) |
| --- | --- | --- | --- | --- | --- | --- | --- |
| Heinrich Heine University Düsseldorf (PD) | Siemens Trio 3T | 2300 | 2.98 | 9 | 192 | 9 | 1 x 1 x 1 |
| RWTH Aachen University (PD) | Siemens Prisma 3T | 2300 | 2.98 | 9 | 176 | 9 | 1 x 1 x 1 |
| Center for Biomedical Research Excellence (SCZ) | Siemens Trio 3T | 2.53 | [1.64, 3.5, 5.36, 7.22, 9.08] | 7 | 192 | 7 | 1 x 1 x 1 |
| University of Groningen (SCZ) | Philips Achieva 3T | 2500 | 4.6 | 30 | 160 | 30 | 1 x 1 x 1 |
| University of Lille (SCZ) | Philips Achieva 3T | 10 | 4.6 | N.A | 160 | N.A | 1 x 1 x 1 |
| Technical University of Munich (SCZ) | Philips Achieva 3T | 9 | 4 | 58 | 170 | 58 | 1 x 1 x 1 |
| Utrecht University (SCZ) | Philips Achieva 3T | 9.86 | 4.6 | N.A | 160 | N.A | 0.875 x 0.875 x 0.875 |

Note: TR, repetition time; TE, echo time; FA, flip angle; SCZ, Schizophrenia; PD, Parkinson’s disease.

**3. Cluster selection criteria**

*Percentage of misclassified voxels*

This criterion indirectly reflects the amount of noise and potentially local effects in the clustering. In particular, the criterion addresses the across-subject stability, that is, the average percentage of voxels for each subject that were assigned to a different cluster compared to the most frequent assignment of these voxels across all subjects. A *k* solution is considered stable if the percentages of deviants are not significantly increased compared to the *k*-1 solution, in particular if the subsequent *k*+1 solution results in a significantly higher percentage of deviants.

*Hierarchy index*

The hierarchy index reflects the percentage of voxels not related to the dominant parent cluster compared to the previous (*k*–1) solution. It corresponds to the percentage of lost voxels that are not present in hierarchy for the *k* solution, compared to the *k*-1 solution. Stable solutions are those *k* solutions where the percentage of lost voxels was below the median across all possible cluster solutions (from 2 to 7 in the current study), where the respective clustering step results in a local minimum and/or the subsequent *k*+1 cluster solution leads to a maximum in the percentage of lost (hierarchically inconsistent) voxels.

*Variation of information across clusters*

The variation of information metric has been previously used to determine the optimal *k*-means parcellation of a given brain region by Kelly et al. (2010) and by Kahnt et al. (2012). This metric was computed for each cluster solution. Solutions with *k* clusters that show a significant increase in variation of information for the subsequent set of solutions with a higher number of clusters or a significant decrease from the previous solution are selected.

*Change in inter/intra cluster distance*

The inter / intra cluster ratio (Chang et al. 2009) calculates the ratio between the average distance of a voxel to its cluster center and the average distance between the cluster centers. The higher the ratio, the better is the separation, a significant increased ratio compared to the previous *k*-1 solution would indicate a better separation of the obtained clusters. However, because of the monotonous increase usually observed with this ratio, we used the first derivative to evaluate the change in this ratio to the previous *k*-1 solution. Stable solutions are those where the subsequent *k*+1 solution does not show a significantly larger increase compared to the current cluster solution.

*Silhouette index*

This criterion measures how similar a voxel is to voxels within its own cluster compared to voxels in other clusters. The silhouette index ranges from -1 to +1. A two-sample *t*-test was used to test for a significant difference in the silhouette value between the current *k* solution and the *k*-1 solution. Stable solutions are those *k* solutions with a significantly higher silhouette value compared to the *k*-1 solution or whose silhouette value is at least not significantly decreased compared to the previous *k*-1 solution.

**4. Regional map (RM) of human and macaque**


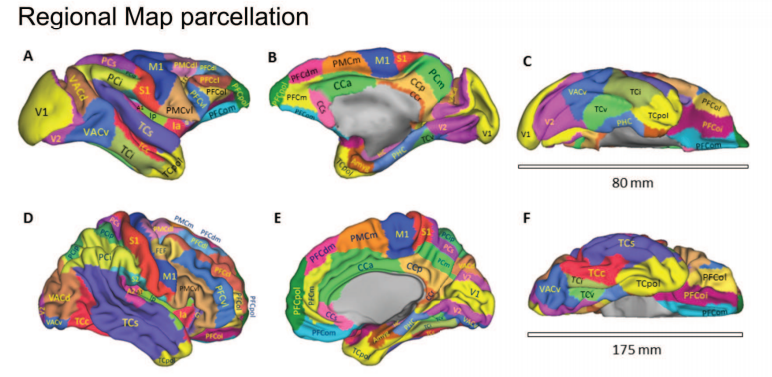


**Fig. S1.** 82 cortical regions from the Region Regional Map (RM) parcellation (right hemisphere), shown on the macaque F99 template surface (A, B and C), and on the human ICBM-152 template surface (D, E and F) (Reid et al. 2016).

**Table S3.** Area names of human and macaque Regional Map (RM).

| **Area name** | **RM** **abbr.** |
| --- | --- |
| Temporal polar cortex | TCpol |
| Superior temporal cortex | STC |
| Amygdala | Amyg |
| Orbitoinferior prefrontal cortex | oiPFC |
| Anterior insula | AI |
| Orbitomedial prefrontal cortex | omPFC |
| Central temporal cortex | CTC |
| Orbitolateral prefrontal cortex | olPFC |
| Inferior temporal cortex | ITC |
| Parahippocampal cortex | PHC |
| Gustatory cortex | GC |
| Ventrolateral premotor cortex | vlPMC |
| Anterior visual area, ventral part | vVAC |
| Posterior insula | PI |
| Prefrontal polar cortex | PFCpol |
| Hippocampus | HC |
| Subgenual cingulate cortex | SCC |
| Ventrolateral prefrontal cortex | vlPFC |
| Visual area 2 (secondary visual cortex) | V2 |
| Medial prefrontal cortex | MPFC |
| Ventral temporal cortex | VTC |
| Anterior visual area, dorsal part | dVAC |
| Visual area 1 (primary visual cortex) | V1 |
| Centrolateral prefrontal cortex | clPFC |
| Secondary auditory cortex | A2 |
| Retrosplenial cingulate cortex | RCC |
| Posterior cingulate cortex | PCC |
| Anterior cingulate cortex | ACC |
| Secondary somatosensory cortex | S2 |
| Primary somatosensory cortex | S1 |
| Primary auditory cortex | A1 |
| Primary motor cortex | M1 |
| Inferior parietal cortex | IPC |
| Medial parietal cortex | MPC |
| Dorsomedial prefrontal cortex | dmPFC |
| Intra-parietal cortex | i-PC |
| Superior parietal cortex | SPC |
| Frontal eye field | FEF |
| Dorsolateral prefrontal cortex | dlPFC |
| Medial premotor cortex | MPMC |
| Dorsolateral premotor cortex | dlPMC |

**5.** **Temporal signal-to-noise (tSNR) results**


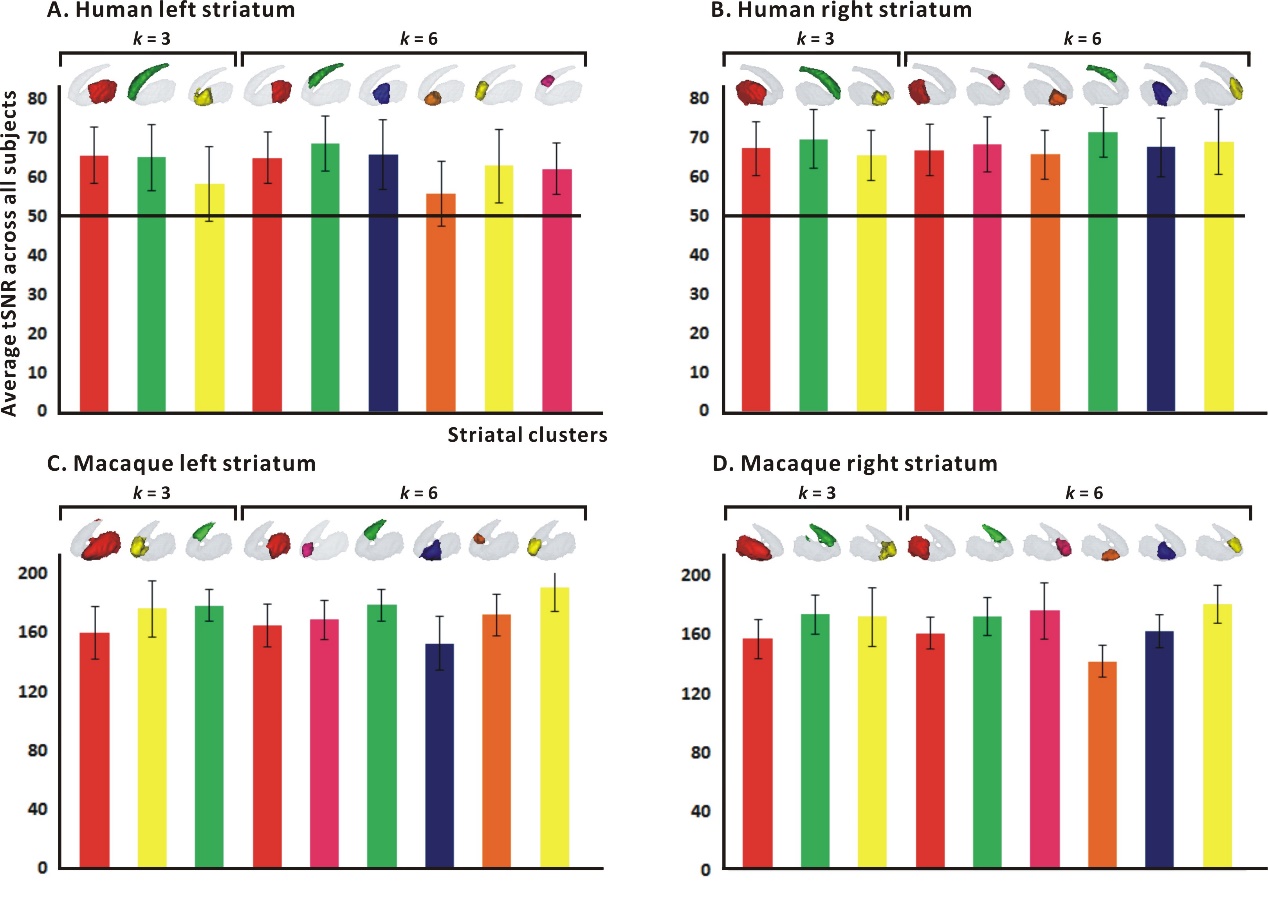
**Fig. S2.** Temporal signal-to-noise (tSNR) of human and macaque striatal clusters across all subjects. The error bars show the variance across subjects.

**6. Results of replication analysis**

Split-half:

We also performed split-half analysis (Strother et al. 2002; LaConte et al. 2003) to assess the robustness of the selected cluster solutions. In this analysis the subjects were randomly split into two halves and the corresponding group-level parcellations were compared using the ARI metric. This analysis was performed for humans and macaques separately. We found that the averaged ARI across 1,000 random split-half analysis was 0.91 (*k* = 3) and 0.89 (*k* = 6) for the human left striatum, 0.91 (*k* = 3) and 0.87 (*k* = 6) for the human right striatum, 0.69 (*k* = 3) and 0.73 (*k* = 6) for the macaque left striatum, and 0.60 (*k* = 3) and 0.73 (*k* = 6) for the macaque right striatum. These results suggested a moderately high-to-high level of match for striatal clusters between halves in split-half analysis.

In addition, we tested the effect of the anesthetized state of macaques on clustering results. We excluded 6 awake macaque subjects from the Newcastle University and examined the reproducibility of striatal clusters. The ARI between the reported clusters and only-anesthetized clusters was 0.97 (*k* = 3) and 0.90 (*k* = 6) for the macaque left striatum, and 0.96 (*k* = 3) and 0.95 (*k* = 6) for the macaque right striatum. These high correspondence shows the robustness of our macaque clustering solutions.

Replication:

We performed two replication analysis for the functional parcellation of human striatum. First using the clinical data from 6 different sites and different demographics (N = 136, age = 33.82 ± 11.11) and the second using an addition sample from the HCP data (N = 206, age = 28.29 ± 3.35). We then estimated the ARI between the original and replicated striatal clusters, the two selected cluster solutions are shown in bold.

Clinical data replication ARI *k* = 2-7: left striatum 0.92, **0.87**, 0.71, 0.68, **0.80**, 0.58, and right striatum 0.84, **0.79**, 0.72, 0.72, **0.76**, 0.74.

HCP replication *k* = 2-7: left striatum 0.85, **0.56**, 0.70, 0.62, **0.81**, 0.58, and right striatum 0.81, **0.85**, 0.77, 0.58, **0.79**, 0.59.

These results show a high level of replicability of our human striatum clustering including for the 3 and 6 cluster solutions which were selected using data-driven criteria.
For macaque we used 12 subjects from the PRIME-DE which were not part of the main sample. The ARI between the replication and reported clustering results for *k* = 2-7 was: left striatum 0.34, **0.31**, 0.52, 0.38, **0.46** and 0.32, and for right striatum 0.02, **0.39**, 0.40, 0.40, **0.42** and 0.40.

In the replication analysis the model selection criterion suggested solutions with 3 and 6 clusters for humans and *k* = 3, 5 and 6 for macaques. Of note, the two selected solutions (*k* = 3 and *k* = 6) generally showed a high ARI.

**Table S4.** The reproducible results by using another dataset, including 206 unrelated subjects from HCP. Abbreviation; ARI: adjusted rand index.

| Hemisphere | ARI | | | | | |
| --- | --- | --- | --- | --- | --- | --- |
|  | *k* = 2 | *k* = 3 | *k* = 4 | *k* = 5 | *k* = 6 | *k* = 7 |
| Left | 0.85 | 0.56 | 0.70 | 0.62 | 0.81 | 0.58 |
| Right | 0.81 | 0.85 | 0.77 | 0.58 | 0.79 | 0.59 |


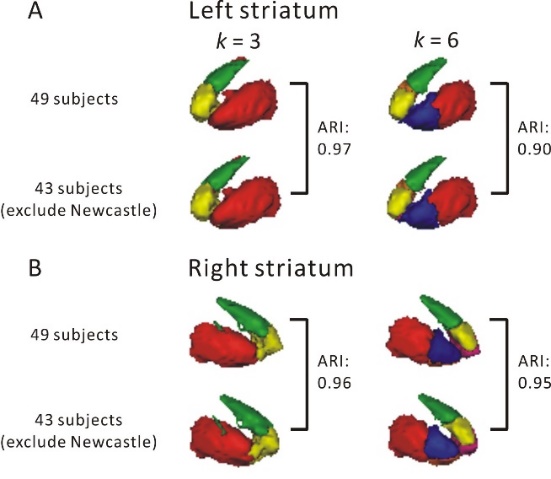


**Fig. S3.** Macaque striatal clusters after excluding the awake subjects from the Newcastle University.

**7. Results of cluster solution criteria**


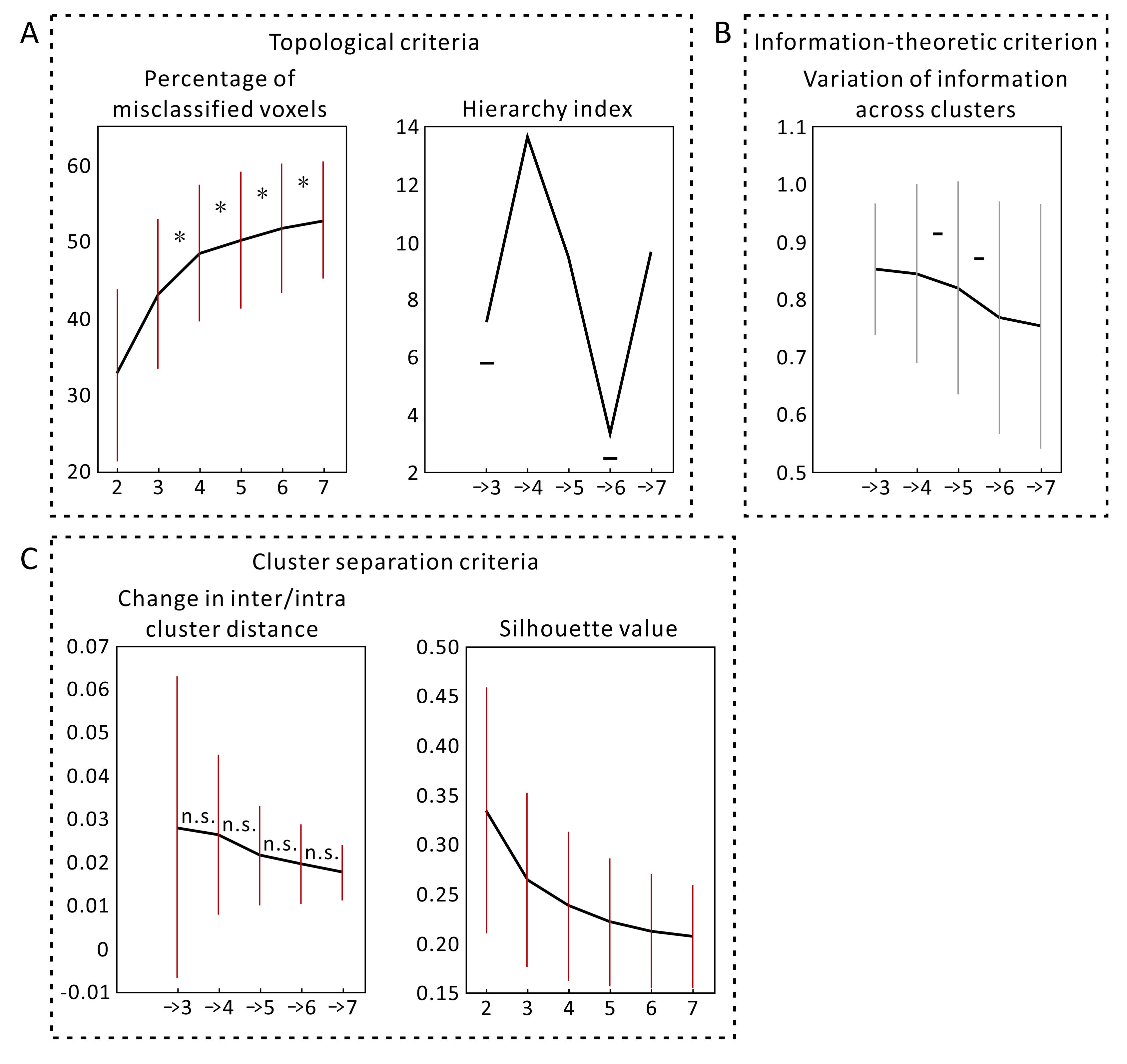


**Fig. S4.** Cluster solution criteria for human left striatum. *Significant change in percentage of deviants. N.s. no significant change.


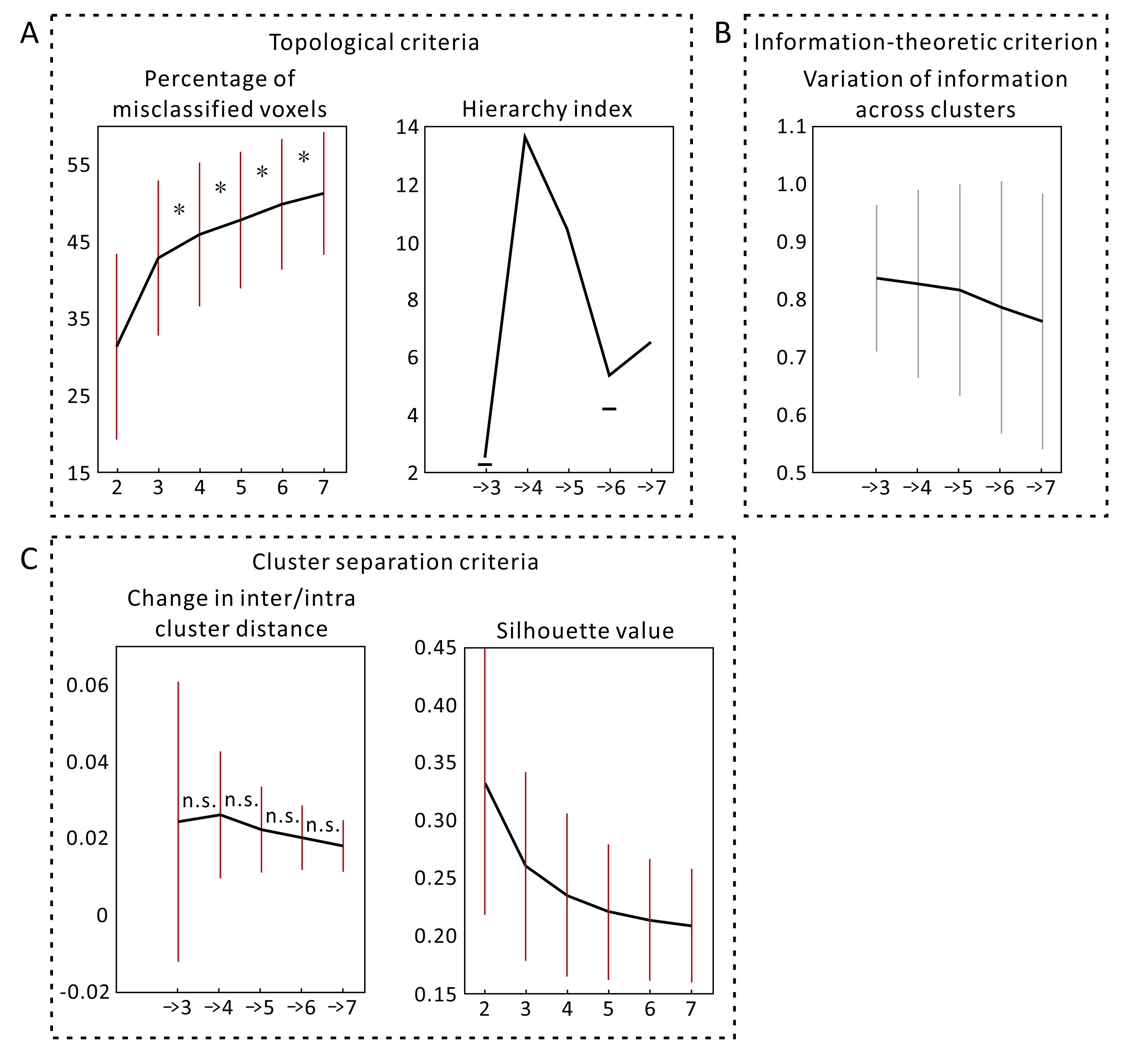


**Fig. S5.** Cluster solution criteria for human right striatum. *Significant change in percentage of deviants. N.s. no significant change.


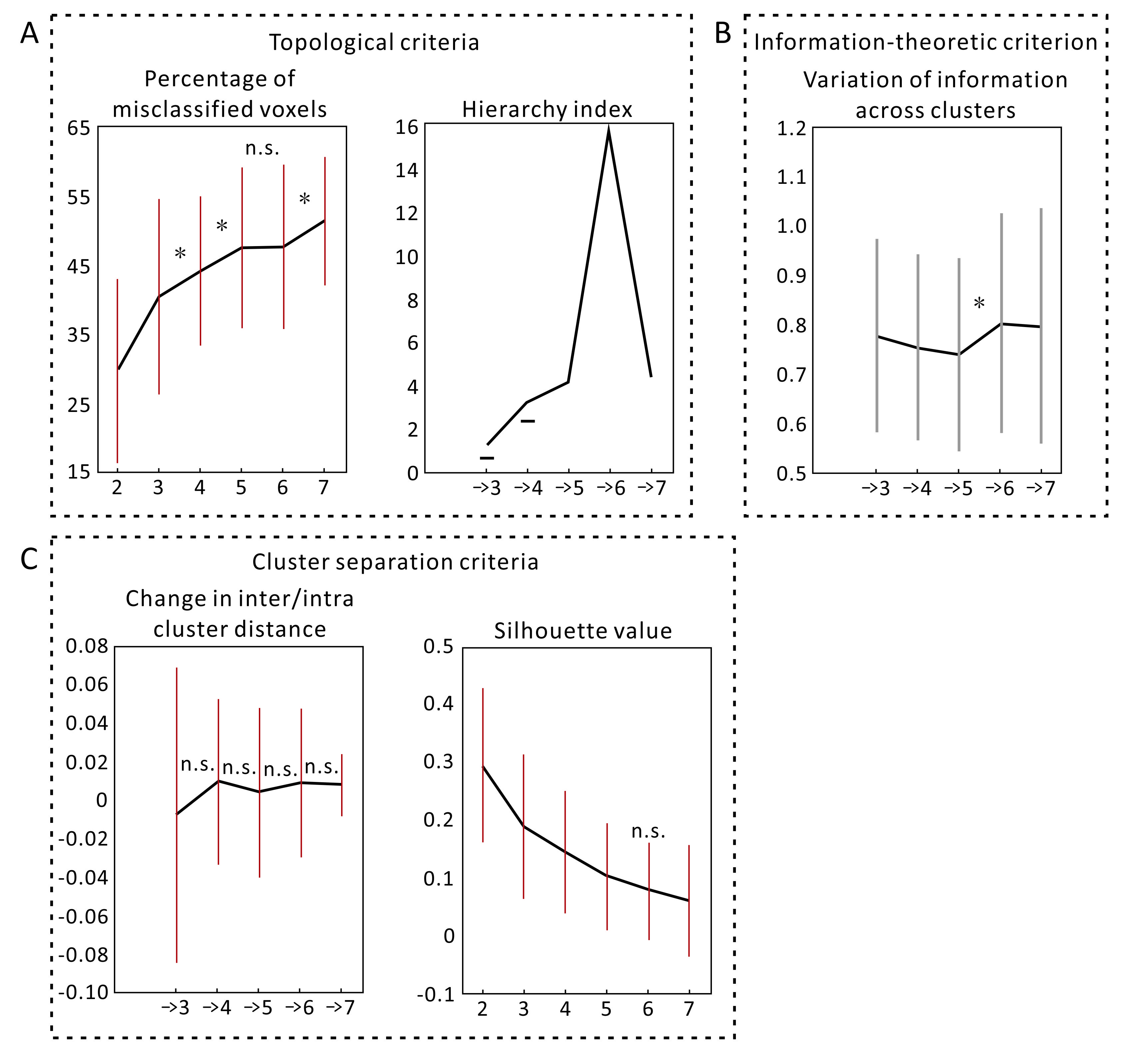


**Fig. S6.** Cluster solution criteria for macaque left striatum. *Significant change in percentage of deviants. N.s. no significant change.


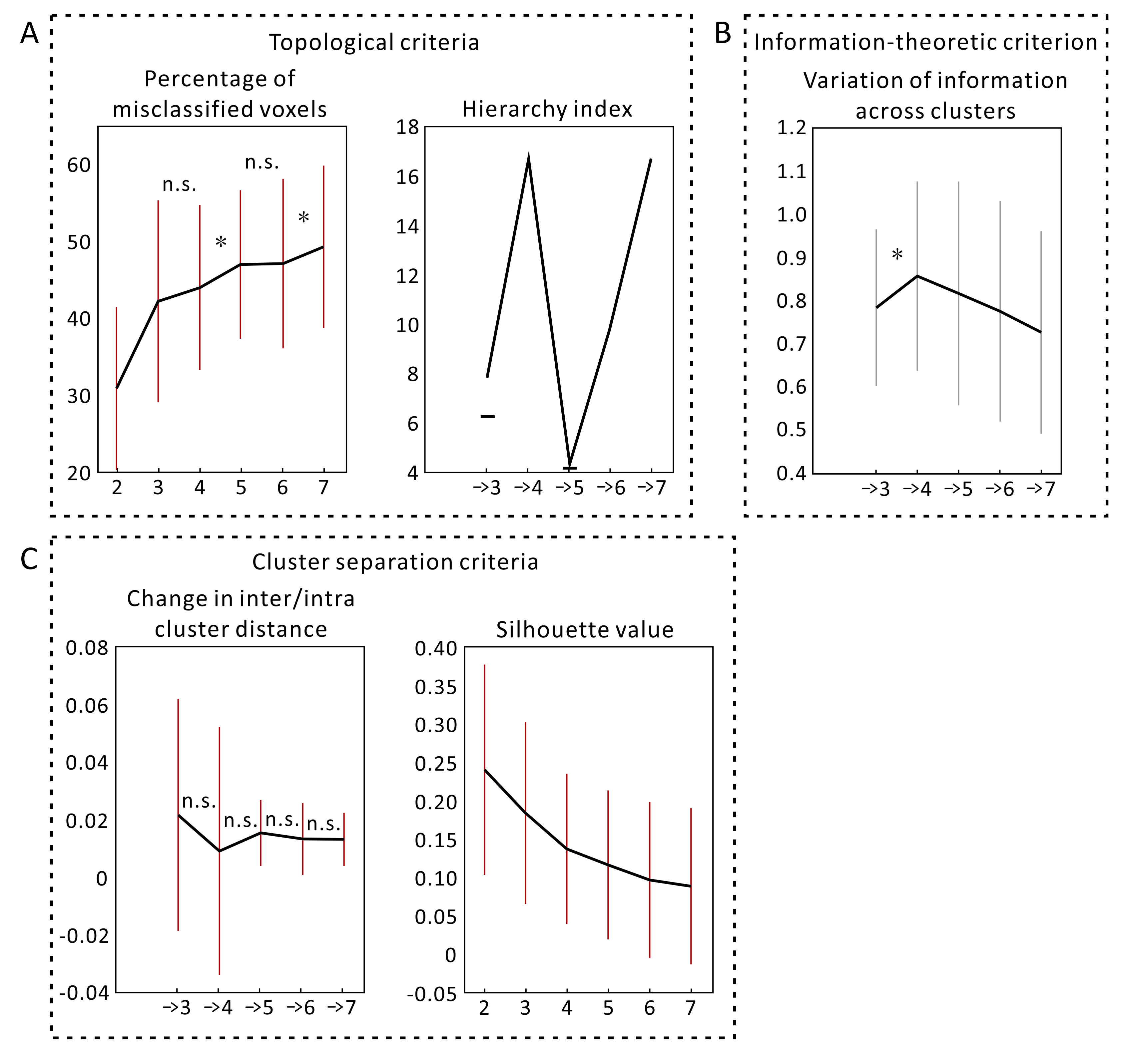


**Fig. S7.** Cluster solution criteria for macaque right striatum. *Significant change in percentage of deviants. N.s. no significant change.

**Table S5.** Cluster selection criteria for human and macaque striatum. The selected solutions using each criterion are shown in bold typeface. Each criterion for selecting stable *k* solution can be found in the section *‘3. Cluster selection criteria’* (Supplementary Materials).

| Criteria | *k*-means cluster solutions | | | | | |
| --- | --- | --- | --- | --- | --- | --- |
|  | *k* = 2 | *k* = 3 | *k* = 4 | *k* = 5 | *k* = 6 | *k* = 7 |
| *Percentage of misclassified voxels* | | | | | | |
| Human left striatum | 32.652 | 43.225 | 48.516 | 50.210 | 51.711 | 52.752 |
| Human right striatum | 31.372 | 42.874 | 45.968 | 47.812 | 49.889 | 51.295 |
| Macaque left striatum | 29.856 | 40.627 | 44.308 | 47.711 | **47.789** | 51.537 |
| Macaque right striatum | 31.068 | 42.278 | 44.103 | 47.100 | **47.241** | 49.417 |
| *Hierarchy index* | | | | | | |
| Human left striatum |  | **7.183** | 13.672 | 9.363 | **3.297** | 9.659 |
| Human right striatum |  | **2.457** | 13.686 | 10.409 | **5.320** | 6.422 |
| Macaque left striatum |  | **1.268** | 3.303 | 4.175 | 15.842 | 4.471 |
| Macaque right striatum |  | **7.839** | 16.805 | 4.239 | 9.896 | 16.763 |
| *Variation of information across clusters* | | | | | | |
| Human left striatum |  | 0.853 | 0.844 | 0.820 | **0.768** | 0.754 |
| Human right striatum |  | 0.837 | 0.827 | 0.817 | 0.786 | 0.762 |
| Macaque left striatum |  | 0.778 | 0.755 | **0.741** | 0.804 | 0.797 |
| Macaque right striatum |  | **0.784** | 0.856 | 0.817 | 0.775 | 0.727 |
| *Change in inter/intra cluster distance* | | | | | | |
| Human left striatum |  | 0.028 | 0.026 | 0.022 | 0.020 | 0.018 |
| Human right striatum |  | 0.024 | 0.026 | 0.022 | 0.020 | 0.018 |
| Macaque left striatum |  | -0.008 | 0.010 | 0.004 | 0.009 | 0.008 |
| Macaque right striatum |  | 0.022 | 0.009 | 0.015 | 0.014 | 0.013 |
| *Silhouette value* | | | | | | |
| Human left striatum | 0.335 | 0.265 | 0.238 | 0.222 | 0.213 | 0.207 |
| Human right striatum | 0.333 | 0.260 | 0.235 | 0.221 | 0.214 | 0.208 |
| Macaque left striatum | 0.296 | 0.189 | 0.144 | 0.103 | **0.079** | 0.062 |
| Macaque right striatum | 0.244 | 0.185 | 0.139 | 0.118 | **0.098** | 0.090 |

**8. CBP robustness checks**


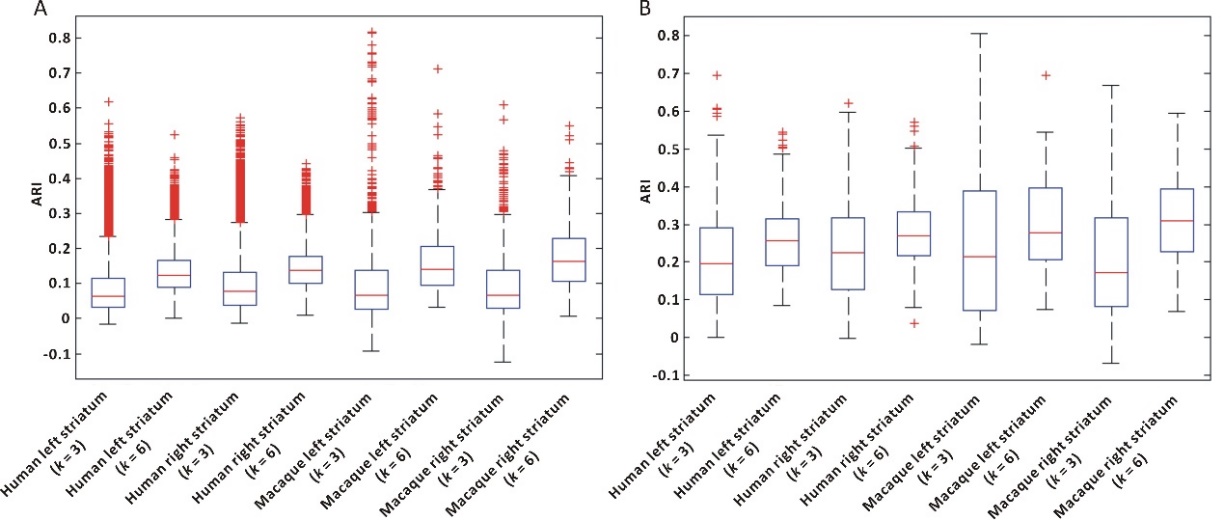


**Fig. S8.** Comparison of A) between-individual cluster solutions (*k* = 3 and *k* = 6) B) individual-level and group-level cluster solutions (*k* = 3 and *k* = 6) by calculating adjusted rand index (ARI).

We performed additional analyses to check the robustness of the group-level CBP clustering solutions.

1) In addition to obtaining the group-level parcellation based on individual-level clustering solutions, we now tested another method where we averaged the connectivity matrices across all subjects and applied *k*-means with desired number of clusters to obtain new group-level parcellation. The ARI between the new and original group-level parcellation: for human striatum 3- and 6-cluster solution: left striatum 0.65 and 0.69, respectively and right striatum 0.62 and 0.67. For macaque striatum 3- and 6-cluster solution: left striatum 0.59 and 0.47, and right striatum 0.74 and 0.54.

2) We performed a permutations analysis to examine whether the ARI between the real group-level and individual-level cluster solutions (*k* = 3 and 6) are significantly better than chance. For this, we shuffled the group-level cluster ids and calculated the ARI between real individual-level clusters and permuted group-level clustering. This was repeated 5,000 times and each time we retained the highest group-to-individual ARI value to obtain the maximum value null distribution. The highest ARI between shuffled group-level and real individual-level clusters ranged from 0.0007 to 0.0392. Compared to the ARI between real group-level and individual-level clusters (0.2-0.3), these results suggested that our group-level clustering are significantly better than chance.

3) We hypothesized that “border-effects” may have reduced individual-to-group ARI values. That is, the cluster assignment of the voxels close to the boundary between clusters are particularly noisy causing lower ARI values. To test this, we removed the border voxels from the group-level clustering and calculated the ARI for the remaining voxels between group-level and individual-level cluster solutions (*k* = 3 and 6). The results show an increase in ARI between 8%-29% (Table S6). Next, to confirm that this increase in ARI is really due to border removal, we removed the same number of voxels as border voxels but now selected randomly from the whole ROI and calculated the group-to-individual ARI. We repeated this random selection 5,000 times to obtain the null distribution and used it to calculate *Z*-scores. All the *Z*-scores were higher than 8.0, confirming that the border voxels bring noise that contributes to lower ARI values.

**Table S6.** Comparison of adjusted rand index (ARI) between individual-level and group-level cluster solutions (*k* = 3 and *k* = 6) when using original and border removed voxels.

| ROI | Original | Border removed | *Z*-score | % increase | #voxels removed |
| --- | --- | --- | --- | --- | --- |
| Human Left (*k*=3) | 0.1996 | 0.2195 | 8.1111 | 9.97 | 198 |
| Human Left (*k*= 6) | 0.2584 | 0.3016 | 14.8947 | 16.72 | 434 |
| Human Right (*k*=3) | 0.2262 | 0.2537 | 10.2790 | 12.16 | 179 |
| Human Right (*k*=6) | 0.2692 | 0.3298 | 19.5546 | 22.51 | 447 |
| Macaque Left (*k*=3) | 0.2134 | 0.2305 | 4.7153 | 8.01 | 272 |
| Macaque Left (*k*=6) | 0.2777 | 0.3466 | 12.0740 | 24.81 | 503 |
| Macaque Right (*k*=3) | 0.1724 | 0.2231 | 7.9490 | 29.41 | 334 |
| Macaque Right (*k*=6) | 0.3105 | 0.3890 | 13.6805 | 25.28 | 519 |


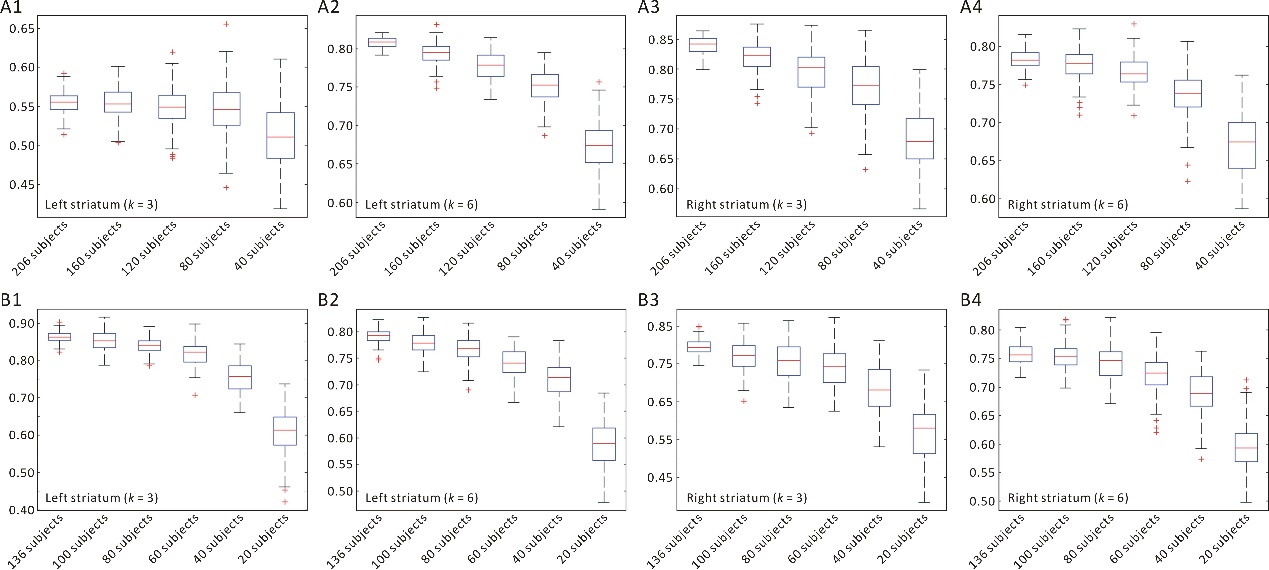


**Fig. S9.** Comparison of group-level clusters between original sample and (A1-A4) HCP replication sample, (B1-B4) clinical sample with different sample sizes by calculating adjusted rand index (ARI).

**9. RSFC between striatal clusters and 7 cerebral networks**

To investigate the functional connectivity pattern of our striatal clusters, we estimated the RSFC between the current striatal clusters (at *k* = 6 solutions) and 7 cortical networks (Yeo et al. 2011). The averaged RSFC between each striatal cluster and cortical network (visual, somatomotor, dorsal attention, ventral attention, limbic, frontoparietal and default mode) across all subjects are show in Fig. S10**.** In comparison with Choi et al. (2012), we found similar RSFC pattern between striatal clusters and 7 cortical networks. This control analysis suggests that our RSFC based parcellation is in-line with a previous study with respect to the connectivity of the parcels with a well-established whole-brain network.


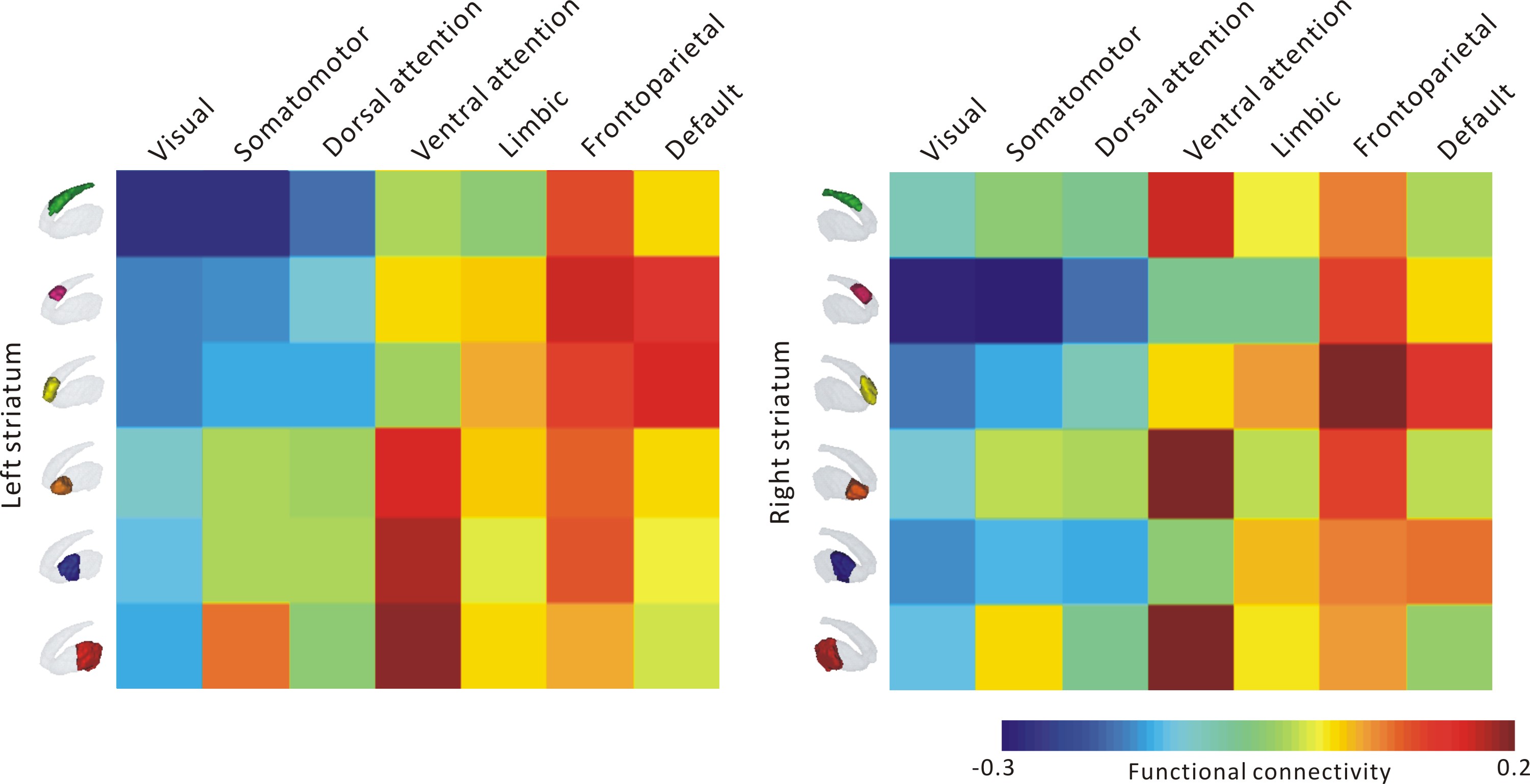


**Fig. S10.** The RSFC between human striatal clusters and 7 cortical networks across all subjects.

**10. CBP using different thresholds of edges in the connectivity matrix**

To check whether thresholding edges in the individual connectivity matrices provide improved group-level signal we reran the CBP analyses at different thresholds with 5%, 25% or 50% of the edges with low connectivity values removed.


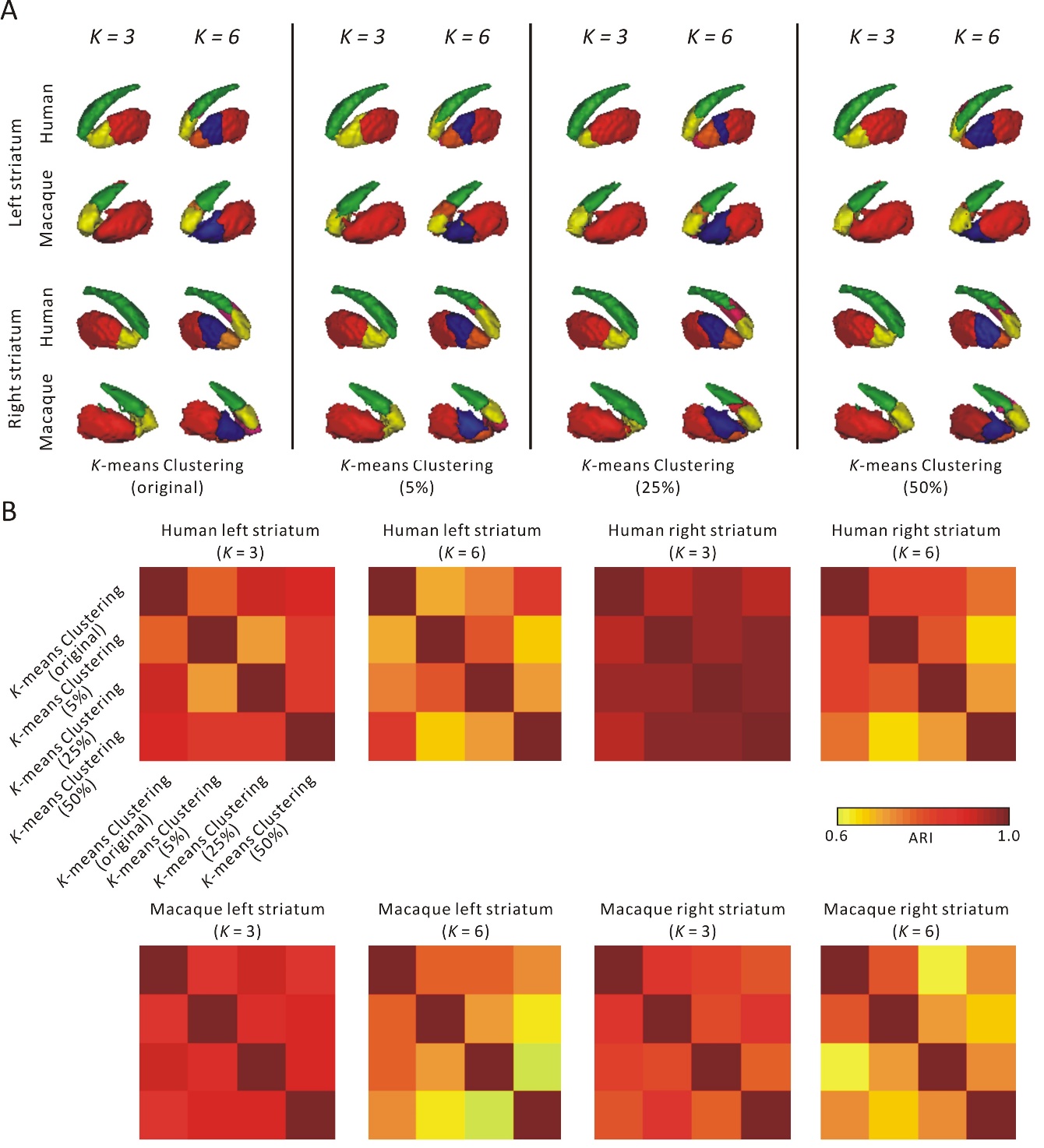


**Fig. S11.** A) Functional parcellation of human and macaque striatum (*k* = 3 and *k* = 6) by using different thresholds of edges in the connectivity matrix. B) Comparison of adjusted rand index (ARI) between any two group-level clusters from different thresholds of edges in the connectivity matrix.

**Table S7.** Comparison of adjusted rand index (ARI) between individual-level and group-level cluster solutions (*k* = 3 and *k* = 6).

| ROI | *K-*means  (original) | *K-*means  (5%) | *K-*means  (25%) | *K-*means  (50%) |
| --- | --- | --- | --- | --- |
| Human Left (*k*=3) | 0.1996 | 0.1812 | 0.1967 | 0.1808 |
| Human Left (*k*=6) | 0.2584 | 0.2346 | 0.2397 | 0.2305 |
| Human Right (*k*=3) | 0.2262 | 0.2150 | 0.2073 | 0.2018 |
| Human Right (*k*=6) | 0.2692 | 0.2658 | 0.2573 | 0.2509 |
| Macaque Left (*k*=3) | 0.2134 | 0.1962 | 0.2104 | 0.1838 |
| Macaque Left (*k*=6) | 0.2777 | 0.2597 | 0.2669 | 0.2733 |
| Macaque Right (*k*=3) | 0.1724 | 0.1683 | 0.1646 | 0.1512 |
| Macaque Right (*k*=6) | 0.3105 | 0.3028 | 0.2992 | 0.3063 |

**References:**

Chang S-E, Kenney MK, Loucks TM, Poletto CJ, Ludlow CL (2009) Common neural substrates support speech and non-speech vocal tract gestures. Neuroimage 47 (1):314-325

Choi EY, Yeo BT, Buckner RL (2012) The organization of the human striatum estimated by intrinsic functional connectivity. Journal of neurophysiology 108 (8):2242-2263

Esteban O, Birman D, Schaer M, Koyejo OO, Poldrack RA, Gorgolewski KJ (2017) MRIQC: Advancing the automatic prediction of image quality in MRI from unseen sites. PloS one 12 (9)

Kahnt T, Chang LJ, Park SQ, Heinzle J, Haynes J-D (2012) Connectivity-based parcellation of the human orbitofrontal cortex. Journal of Neuroscience 32 (18):6240-6250

Kelly C, Uddin LQ, Shehzad Z, Margulies DS, Castellanos FX, Milham MP, Petrides M (2010) Broca’s region: linking human brain functional connectivity data and non‐human primate tracing anatomy studies. European Journal of Neuroscience 32 (3):383-398

LaConte S, Anderson J, Muley S, Ashe J, Frutiger S, Rehm K, Hansen LK, Yacoub E, Hu X, Rottenberg D (2003) The evaluation of preprocessing choices in single-subject BOLD fMRI using NPAIRS performance metrics. NeuroImage 18 (1):10-27

Magnotta VA, Friedman L, Birn F (2006) Measurement of signal-to-noise and contrast-to-noise in the fBIRN multicenter imaging study. Journal of digital imaging 19 (2):140-147

Power JD, Barnes KA, Snyder AZ, Schlaggar BL, Petersen SE (2012) Spurious but systematic correlations in functional connectivity MRI networks arise from subject motion. Neuroimage 59 (3):2142-2154

Reid AT, Lewis J, Bezgin G, Khundrakpam B, Eickhoff SB, McIntosh AR, Bellec P, Evans AC (2016) A cross-modal, cross-species comparison of connectivity measures in the primate brain. Neuroimage 125:311-331

Strother SC, Anderson J, Hansen LK, Kjems U, Kustra R, Sidtis J, Frutiger S, Muley S, LaConte S, Rottenberg D (2002) The quantitative evaluation of functional neuroimaging experiments: the NPAIRS data analysis framework. NeuroImage 15 (4):747-771

Yeo BT, Krienen FM, Sepulcre J, Sabuncu MR, Lashkari D, Hollinshead M, Roffman JL, Smoller JW, Zöllei L, Polimeni JR (2011) The organization of the human cerebral cortex estimated by intrinsic functional connectivity. Journal of neurophysiology
